# SPARC: a structural pathogenicity algorithm for risk classification of hERG variants

**DOI:** 10.1093/europace/euaf327

**Published:** 2025-12-25

**Authors:** Frank C Chatelain, Barbara Ribeiro de Oliveira, Guillaume Grataloup, Noé Robert, Malak Alameh, Aurélie Thollet, Jérôme Montnach, Sylvain Feliciangeli, Aline Rio, Floriane Bibault, Delphine Bichet, Olivier Bignucolo, Fabrice Extramiana, Rupamanjari Majumder, Jean-Jacques Schott, Vincent Probst, Isabelle Denjoy, Florian Lesage, Gildas Loussouarn, Michel De Waard

**Affiliations:** Université Côte d’Azur, INSERM, CNRS, Institut de Pharmacologie Moléculaire et Cellulaire, Valbonne, France; LabEx « Ion Channels, Science and Therapeutics », Université Côte d'Azur, Valbonne, France; LabEx « Ion Channels, Science and Therapeutics », Université Côte d'Azur, Valbonne, France; Nantes Université, CNRS, INSERM, l’institut du thorax, Nantes F-44000, France; Université Côte d’Azur, INSERM, CNRS, Institut de Pharmacologie Moléculaire et Cellulaire, Valbonne, France; Université Côte d’Azur, INSERM, CNRS, Institut de Pharmacologie Moléculaire et Cellulaire, Valbonne, France; LabEx « Ion Channels, Science and Therapeutics », Université Côte d'Azur, Valbonne, France; Nantes Université, CNRS, INSERM, l’institut du thorax, Nantes F-44000, France; CHU Nantes, l’institut du thorax, INSERM, CNRS, UNIV Nantes, Nantes, France; LabEx « Ion Channels, Science and Therapeutics », Université Côte d'Azur, Valbonne, France; Nantes Université, CNRS, INSERM, l’institut du thorax, Nantes F-44000, France; Université Côte d’Azur, INSERM, CNRS, Institut de Pharmacologie Moléculaire et Cellulaire, Valbonne, France; LabEx « Ion Channels, Science and Therapeutics », Université Côte d'Azur, Valbonne, France; Nantes Université, CNRS, INSERM, l’institut du thorax, Nantes F-44000, France; Nantes Université, CNRS, INSERM, l’institut du thorax, Nantes F-44000, France; Université Côte d’Azur, INSERM, CNRS, Institut de Pharmacologie Moléculaire et Cellulaire, Valbonne, France; Université Côte d’Azur, INSERM, CNRS, Institut de Pharmacologie Moléculaire et Cellulaire, Valbonne, France; Département de Rythmologie, Centre de Référence Maladies Cardiaques Héréditaires, Filière Cardiogen, Paris, France; Nantes Université, CNRS, INSERM, l’institut du thorax, Nantes F-44000, France; Nantes Université, CNRS, INSERM, l’institut du thorax, Nantes F-44000, France; CHU Nantes, l’institut du thorax, INSERM, CNRS, UNIV Nantes, Nantes, France; Département de Rythmologie, Centre de Référence Maladies Cardiaques Héréditaires, Filière Cardiogen, Paris, France; Université Côte d’Azur, INSERM, CNRS, Institut de Pharmacologie Moléculaire et Cellulaire, Valbonne, France; LabEx « Ion Channels, Science and Therapeutics », Université Côte d'Azur, Valbonne, France; LabEx « Ion Channels, Science and Therapeutics », Université Côte d'Azur, Valbonne, France; Nantes Université, CNRS, INSERM, l’institut du thorax, Nantes F-44000, France; Nantes Université, CNRS, INSERM, l’institut du thorax, Nantes F-44000, France

**Keywords:** hERG channel, Arrhythmia, Variant of uncertain significance, Structural pathogenicity score, High throughput phenotyping, UCSF Chimera

## Abstract

Inherited mutations in the *KCNH2* gene, which encodes the cardiac hERG potassium channel, are major contributors to arrhythmogenic syndromes such as long QT and short QT syndromes. However, clinical interpretation of the growing number of missense variants – many of which are classified as variants of uncertain significance (VUS) – remains a pressing challenge. Here, we present a semi-automated *in silico* pipeline for predicting hERG variant pathogenicity, acting as a binary classifier and integrating five structural metrics – residue volume, hydrophobicity, charge, steric clashes, and proximity to pathogenic hotspots – into a composite structural pathogenicity score (SPS) scaled from 1 to 5. Applied to 1727 hERG variants from ClinVar and from a French nationwide cohort, this binary classifier, termed SPARC, identified 260 variants as high risk of pathogenicity with SPS ≥3.25, of which a representative subset from the French cohort was functionally validated using high-throughput automated patch-clamp. Functional phenotyping confirmed the structural predictions, including for several VUS, demonstrating that comprehensive structural scoring can reliably stratify variant pathogenicity. This approach, benchmarked with Alpha Missense and Revel, offers a superior scalable, cost-effective pre-screening tool to guide clinical variant interpretation and prioritization for experimental validation.

## Graphical Abstract

**Graphical Abstract:**
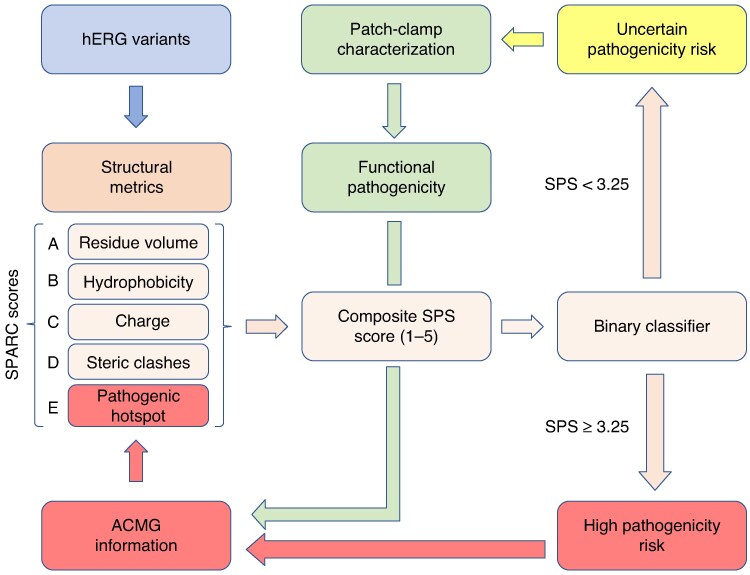

## Introduction

Mutations in the *KCNH2* gene, which encodes the cardiac delayed rectifier potassium channel hERG, are well-established drivers of inherited arrhythmia syndromes.^1^ Loss-of-function mutations typically underlie long QT syndrome (LQTS),^2^ whereas gain-of-function mutations are associated with short QT syndrome (SQTS).^3^ Both conditions are linked to an increased risk of sudden cardiac death due to malignant ventricular arrhythmias, underscoring the clinical imperative to identify individuals at highest risk. With the widespread adoption of high-throughput exome and genome sequencing,^4^ the number of identified *KCNH2* variants has risen dramatically. As of now, 3658 variants have been catalogued in ClinVar (https://www.ncbi.nlm.nih.gov/clinvar/), including 1752 missense variants.^5^ Given that the hERG1a isoform comprises 1159 amino acids, nearly every residue appears susceptible to variation. However, the clinical interpretation of these variants remains a major challenge. The American College of Medical Genetics and Genomics (ACMG), in collaboration with the Association for Molecular Pathology (AMP) and the College of American Pathologists (CAP), established guidelines to systematically evaluate the potential pathogenicity of genetic variants.^6^ These guidelines incorporate a comprehensive set of criteria, including the patient’s clinical phenotype, segregation data, evolutionary conservation of the altered sequence, population frequency data, results from *in vitro* functional assays, the biochemical nature of the substituted amino acids, and the position of the change within the protein. Based on a weighted scoring system, each variant is then classified into one of five levels of certainties: ‘benign’ (referred herein as class 1), ‘likely benign’ (class 2), ‘variant of uncertain significance’ (VUS or class 3), ‘likely pathogenic’ (class 4) or ‘pathogenic’ (class 5). A variant is typically labelled as VUS when either the available data are contradictory across criteria, or the evidence is insufficient – situations frequently encountered with novel missense variants. Indeed, 1406 (80%) of hERG missense variants are currently classified as VUS, including 1242 strict VUS (71%) and 164 (9%) with conflicting interpretations. Other VUS include 10 frameshifts out of 357 (2.8%), 1 nonsense out of 98 (1%), 5 splice sites out of 68 (7.3%), 401 non-coding RNA out of 1193 (33.6%), and 36 UTR out of 95 (37.9%), indicating that the vast majority of VUS is supported by missense.^7,8^ This high proportion of unclassified variants complicates diagnosis, genetic counselling, and patient management. Several initiatives have been launched to classify genetic variants from traditional genetic studies^4,9^ up to more recent machine learning strategies.^10–12^ Alpha Missense^11^ and REVEL^12^ are two of the best-performing machine learning strategies. The first one is based on protein structure, but does not take into account the dynamic interactions between the new residues and those in the immediate vicinity of the substitution, while the second one relies on a combination of 13 different prediction tools. All these approaches agreed on the importance of functional studies to unequivocally conclude about ion channel variant pathogenicity. However, detailed functional assays in heterologous expression systems^13–15^ are labor-intensive, often requiring specialized experimental expertise and access to advanced electrophysiological platforms – resources that are not routinely available to most researchers or clinicians – and thus not inherently cost-efficient. In a previous study^16^ aimed at developing a rapid protocol for variant assessment, we proposed a streamlined structural analysis strategy to predict the pathogenic potential of hERG variants, contingent upon their localization within defined structural domains delineated by the original Cryo-EM structure of the channel.^17^ The regions covered by this structural approach notably exclude substantial portions of both the N-linker and C-terminal domains of hERG. Pathogenicity was assessed using the open-source UCSF Chimera platform,^18^ based on a set of local structural features, embracing spatial residue occupancy, hydrophobic character, and the extent of intermolecular interactions with neighboring residues. While this analysis is constrained to local perturbations in the immediate vicinity of the variant, it yielded a compelling insight: whenever structural criteria predicted pathogenicity, the corresponding variant consistently exhibited functional defects.^16^ Although not exhaustive, this method offers a valuable opportunity to expedite the reclassification of hERG variants as pathogenic, thereby potentially obviating the need for immediate full-scale electrophysiological evaluation. Moreover, it can serve as an initial screening tool to triage variants according to their pathogenic potential before confirmatory functional studies.

Building on this proof of concept, we sought to (i) improve the structural analysis for detecting variant-induced alterations; (ii) develop an automated, open-access algorithm leveraging UCSF Chimera to streamline variant analyses and categorize their pathogenicity according to a binary classifier (high vs. uncertain pathogenic risk) – applicable to other ion channel types; and (iii) validate this classifier by functionally characterizing 33 clinically reported French hERG variants, selected based on either algorithmic scoring or classification under ACMG criteria.^6^ For robust functional validation, we employed an optimized, high-throughput patch-clamp platform capable of capturing a comprehensive suite of hERG biophysical parameters under a fast-track protocol.^16^ Our results demonstrate that this Structural Pathogenicity Algorithm for Risk Classification (SPARC) performs well as a binary classifier: randomly-picked variants with structural pathogenicity scores (SPS) ≥3.25 have a high risk of pathogenicity as assessed by patch-clamp studies, whereas variants with scores <3.25 should be considered of uncertain pathogenic risk. SPARC can be considered a cost- and time-efficient algorithm for prioritizing functional studies of hERG variants that remain of uncertain pathogenic risk.

## Materials and methods

### hERG variants used for structural pathogenicity prediction

For this study, we investigated all the hERG missense variants available in ClinVar^7,8^ (01 August 2025) and in the French Bamacoeur database (from the national network, Cardiogen – http://www.filiere-cardiogen.fr/).^16,19^ This represents a total of 1727 variants (with 358 variants in Bamacoeur, of which 248 are in both databases).^5^ All variants were implemented in the algorithm.

### Update of the ACMG classification

For Bamacoeur *KCNH2* variants, the criteria BS3/PS3 of ACMG required for variant classification were updated by F. Kyndt at the ‘Centre de Références des Maladies Rares’ (Nantes, France) with available functional data retrieved from bibliographic searches and integrated into InterVar^20^ (list of residues available in Supplementary material online, *Table S1*).

### Scoring method for structural alteration produced by hERG variants

**Figure 1:**
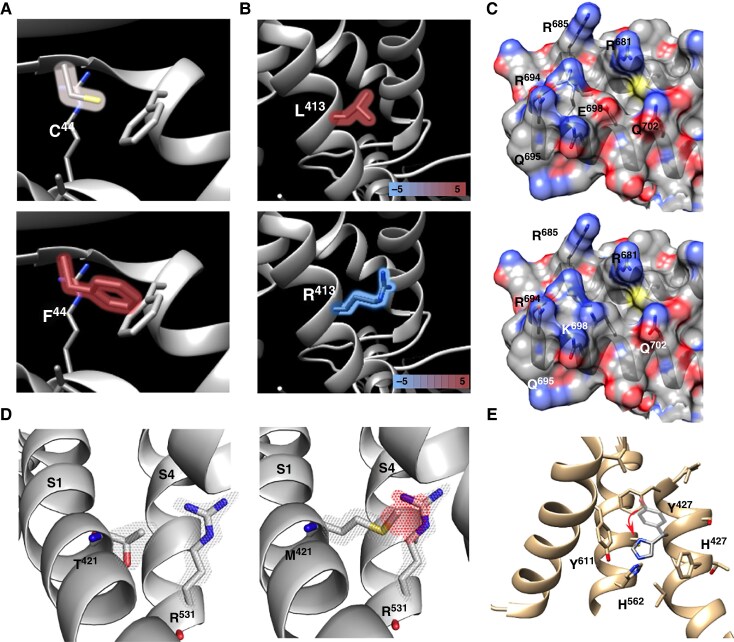
Illustration of Scores A to E. *A*, Score A illustration assessing surface area and volume alteration. For example, the C^44^F mutation yields a median Score A of 0.3798, compiling surface and volume estimations (see Supplementary material online, *Figure S3*), which represents about one-third of the maximum observed difference for amino acid substitutions. *B*, Score B evaluating the magnitude of hydrophobicity change caused by the amino acid substitution, based on a median of four scales (see Supplementary material online, *Figure S6*). In the case of the L^413^R mutation, a median Score B of 0.7861 is assigned, reflecting three-quarters of the maximal possible difference and therefore a substantial alteration in local hydrophobicity. *C*, Score C accounts for the electrostatic consequences of charge inversion at the mutation site. The loss of a negative charge and the gain of a positive charge are each penalized by a semi-point. For instance, the E^698^K mutation, which replaces a negatively charged glutamate with a positively charged lysine, is assigned the maximum Score C of 1, reflecting the full reversal in local electrostatic properties (see Supplementary material online, *Figure S7*). *D*, Score D reflects the probability of a clash between the variant and a residue in close vicinity, as well as the protein's ability to easily accommodate this clash according to the function ‘Minimize Structure’ of the UCSF Chimera software. In this example, the side chain of residue T^421^, located on the S1 domain, is oriented towards the side chain of R^531^ located on the adjacent S4 domain (left panel). According to the Dunbrack library, over 50% of the possible methionine rotamers of the T^421^M variant are associated with clashes with neighboring residues (red mesh, right panel). The probability of occurrence of these clashes and their ability to disappear or not after structure minimization by the UCSF Chimera software, weights the score of this mutation in a range from 0 to 1, here a score of 0.64. *E*, Score E reflects the degree of potential pathogenicity of residues surrounding the variant. Among the various rotamers of the neo-residue that occur with a probability greater than 10%, some come in direct contact with residues known to be potentially pathogenic in other variants. In this example, for the Y^427^H variant (transparent grey), the new histidine residue has two possible rotamers with a probability of occurrence higher than 10%. The first rotamer, with a probability of 20.75%, is in direct contact with 4 neighboring residues, including 1 that is known to be highly pathogenic when mutated (H^562^N or H^562^P). The second rotamer, with a probability of 11.2%, is in direct contact with 5 neighboring residues, including 2 that are highly pathogenic when mutated (H^562^N or H^562^P, and Y^611^H). Hence, Score E is calculated as follows: E = ¼ x 0.2075 + 2/5 × 0.112 = 0.097 (further developed in Supplementary data).

For the purpose of this study, the cryoEM structure of hERG was used (https://doi.org/10.2210/pdb5va1/pdb).^17^ Macros running on the UCSF Chimera software (version 1.16)^18^ were developed to produce scores of variant pathogenicity based on five different criteria: (1) alteration in size induced by the substitution (Score A), (2) change in degree of hydrophobicity (Score B), (3) modification in residue charge (Score C), (4) severity of steric clashes (Score D), and (5) proportion of interacting amino acids in contact with the residue of interest and for which a substitution is pathogenic (Score E) (examples are illustrated in *Figure 1*). Score B and Score C result from the subdivision of previous Score B.^16^ The fifth metric, Score E, is a novel addition in this study, compared to earlier efforts,^16^ and reflects the pathogenic landscape of residues in direct contact with the variant for each significant rotamer. This analysis draws on an extensive body of functional data from published studies,^5^ identifying contact residues that are themselves known to be pathogenic when mutated (classified as ACMG Class 4 or 5). This score thus captures potential pathogenic ‘hotspots’ in the variant’s structural vicinity. Hence, Scores A to D measure predicted severity of change to the hERG channel, while Score E is slightly probabilistic. Of note, Scores A to C are independent of hERG 3D structure, whereas Scores D and E require the 3D structure. For this reason, variants located within amino acid positions 132–398, 433–448, 511–519, 578–582, 598–602, and 864–1159, lacking 3D structure, will benefit only from scoring A to C. Every other variant will benefit from the full range of A to E scoring. The details of the scoring system for each criterion are provided in Supplementary data (Supplementary material online, *Table S2*, *S3*, Supplementary material online, *Figures S1*–*S7*). Briefly, all Scores range from 0 to 1 on a continuous scale, with the exception of Score C, based on charge alteration that takes only values 0, 0.5, and 1.

### Scaling of the final structural pathogenicity score

While the theoretical range for the cumulative pathogenicity score (sum of Scores A through E) spans from 0 to 5, it has been normalized to span the same ACMG 5-tier classification system (scores from 1 to 5). In practice, during the study, observed cumulative values ranged from 0 to 3.30 because none of the variants displayed a score of 1 for all scores A to E. To align these values with the ACMG scale, the SPS A–E was rescaled such that the minimum and maximum values corresponded to 1 and 5, respectively, and all scores were rounded to the nearest 0.25 increment. The transformation applied was as follows:


(1) $$y=1+\frac{\left\lfloor x+\frac{1}{2}\right\rfloor }{4}\;\mathrm{with}\;x=(((A+B+C+D+E)/3.3)x4x4).$$

For unresolved hERG regions, where structural information is lacking, Scores D and E were set to 0, and the same equation was used.

### SPARC: the algorithm that integrates the scoring matrices and performs calculations using the UCSF Chimera for automated prediction of variant pathogenicity

The Python macro that implements the algorithm is available online at https://doi.org/10.5281/zenodo.15422598. It takes into account all our methodology for pathogenicity scoring as described above and integrates a user-defined exploitation of the UCSF Chimera software (https://www.cgl.ucsf.edu/chimera/). Its use generates, in the final run, a Microsoft Excel (version 16.98) sheet that contains the individual scorings (**A** to **E**), the rotamers, the surrounding residues for each rotamer, and the final score of each variant. It also generates individual data sheets with more detailed information for each variant. These sheets are also available at https://doi.org/10.5281/zenodo.15422598. Of note, this algorithm is not only valid for hERG but for all other ion channels whose structures have been determined. It only requires redefining the scaling factor mentioned in equation (1) as well as the PDB file corresponding to the protein studied, the amino acid sequences lacking in the structure, and the non-exhaustive list at the time of the study of the amino acid residues known to be pathogenic.

### Cell culture

HEK293 (ATCC CRL-3216) cells were cultured according to the conditions previously described.^16^

### Plasmid for hERG channel mutagenesis and electroporation conditions

Mutagenesis of the hERG channel (NCBI reference: NM_000238.4), cloned between the *Hind*III and *Xho*I restriction sites of the pCDNA5/FRT/TO plasmid, was performed as previously described using the Gibson assembly method.^16^ One change as compared to our previous work consisted of the removal of the combined C-terminal transmembrane transferrin segment and the pHluorin tag so that only the exact sequence of the hERG channel was expressed. Missense mutations were selected on the basis of structural predictions. All plasmids were introduced into HEK293 cells by electroporation using OC-100 cassettes with the MaxCyte STx system (MaxCyte Inc., MD, USA) as described previously.^21^ Twenty-four hours after transfection, cells were treated with trypsin and resuspended in external NMDG solution (in mM: 80 NaCl, 4 KCl, 2 CaCl_2_, 1 MgCl_2_, 5 glucose, 60 N-Methyl-D-Glucamine (NMDG), 10 HEPES – pH 7.4 (NaOH), 280 ± 3 mOsm) for automated patch-clamp experiments.

### Optimized fast-track and exhaustive protocol for hERG variant evaluation on a high-throughput automated patch-clamp system

hERG biophysical properties were all evaluated in the homozygous condition throughout the manuscript. This was deemed the perfect match to fit with predictions of the structural impact of the variants. Specific attention was carried on French Bamacoeur variants. The protocol used for the Nanion Syncropatch 384PE was adapted from the set of sub-protocols previously used in manual patch-clamp.^16^ Major adaptations were introduced in this fast-track protocol to take into account the constraints of the Nanion’s PatchControl software and the ionic conditions required for making gigaseals with the Syncropatch. This fast-track protocol now contains 6 sub-protocols as defined (see Supplementary material online, *Figure S8*). In addition, external Ca^2+^ levels are higher (2.88 mM as compared to 1 mM with manual patch-clamp) and internal fluoride concentrations are high (110 mM). Holding potential was kept at −80 mV. The durations of the steps were optimized to take into account the voltage-dependence of activation and inactivation kinetics.^16^ The duration of the entire biophysical protocol was 74 s, allowing the extraction of all biophysical parameters (nine in total). These parameters were normalized with regard to wild-type hERG parameters systematically recorded at the same time and represented on radar plots along with the standard deviation of the wild-type parameters. Recordings were performed with external NMDG solution (cf. above) and internal solution (in mM: 110 KF, 10 NaCl, 10 KCl, 10 mM EGTA, 10 HEPES – pH 7.2 (NaOH), 280 ± 3 mOsm).

### Data analyses by R scripts

R automated routines used for analyzing the data generated by automated patch-clamp are all available from a repository https://zenodo.org/records/15847627. The following quality controls were used to exclude cells for data analyses: access resistance Ra > 10 MΩ, seal resistance Rs < 600 MΩ measured at −86 mV, leak currents >200 pA at −60 mV, and run-down >20% measured on I_max_ at 90 mV from beginning to end of the biophysical protocol (74 s). For the extraction of biophysical properties, a minimal current amplitude of 500 pA was required, but not exceeding 10 nA.^22^ hERG channel reversal potential was −66.4 ± 10.4 mV (*n* = 2437 cells). To reduce variability, all variant parameters were compared to those of the wild-type hERG expressed on the same day. The R routines include statistical analyses that are parametric t-tests for amplitudes and biophysical parameters, and ANOVA tests for hERG channel kinetic parameters measured at several potentials. *, *P* < 0.05; **, *P* < 0.01; ***, *P* < 0.001.

## Results

### Distribution of hERG missense variants along the channel structure according to ACMG classification

**Figure 2:**
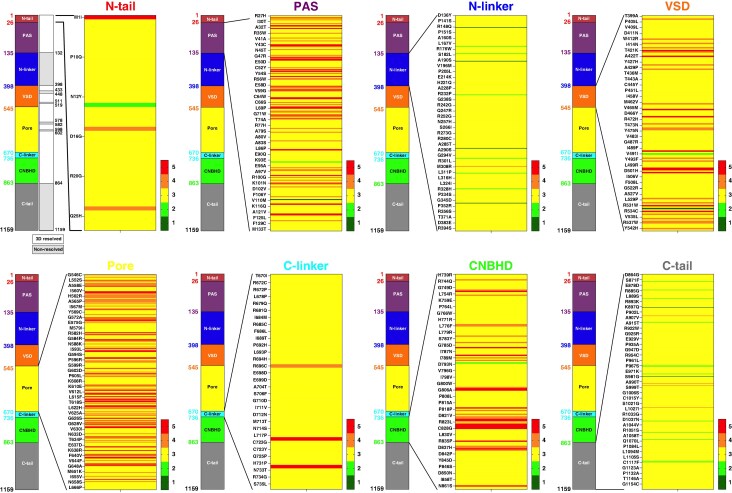
ACMG scoring of 1727 *KCNH2* missense variants as distributed along the structural domains of hERG. Very few benign variants are represented in the ACMG classification, whereas the vast majority are VUS (class 3). Pathogenic variants (classes 4 & 5) represent less than 15% of total. Detailed ACMG scores for each variant are provided in Supplementary material online, *Table S1*.

To assess the relevance of a structural framework in evaluating hERG variant pathogenicity, we analyzed the ACMG classification of all 1727 hERG missense variants, mapped across defined structural domains of the protein (*Figure 2*). The distribution across ACMG categories was as follows: eight variants (0.46%) classified as benign (Class 1), 22 (1.27%) as likely benign (Class 2), 1447 (83.79%) as VUS (Class 3), 151 (8.74%) as likely pathogenic (Class 4), and 99 (5.73%) as pathogenic (Class 5). These proportions indicate that it is easier to classify a variant as pathogenic than benign (only 30 classes 1 or 2 over 1727 missense variants). 24 of the 30 benign variants localize in the non-resolved structural domains (N-linker and C-tail). Notably, 95.6% of all ACMG-classified pathogenic variants (Classes 4 and 5, *n* = 250) localize within four structurally characterized regions: the Per-Arnt-Sim (PAS) domain (64 variants), the voltage-sensing domain (VSD, 30 variants), the pore domain (112 variants), and the cyclic nucleotide-binding homology domain (CNBHD, 33 variants) (see Supplementary material online, *Table S1*). The frequency of pathogenic variants within these domains is substantial: 28.6% in the PAS, 20.0% in the VSD, 39.4% in the pore domain, and 19.6% in the CNBHD. These findings point to the pore domain as being particularly sensitive to sequence alterations in terms of pathogenicity. In contrast, the regions with the lowest incidence of ACMG-classified pathogenic variants are the N-terminal tail, the N- and C-linkers, and the C-terminal tail – segments that include intrinsically disordered regions as previously predicted.^17^ The N-linker has a single pathogenic variant (G^306^W) out of 346 variants identified. These domains thus logically display the highest proportion of VUS: 92.6% in the N-tail (54 total variants), 96.8% in the N-linker (346 total variants), 95.1% in the C-linker (61 total variants), and 95.9% in the C-tail (440 total variants). These findings suggest that such structurally ambiguous regions remain poorly explored and highlight the unmet need for more comprehensive functional and structural analyses. A central aim of our algorithm is to assist in the reclassification of a fraction of all VUS by uncovering latent pathogenicity. It may also serve to corroborate the current ACMG designations for variants already deemed pathogenic (Classes 4 and 5).

### Predicting hERG variant pathogenicity via multi-parametric structural scoring

**Figure 3:**
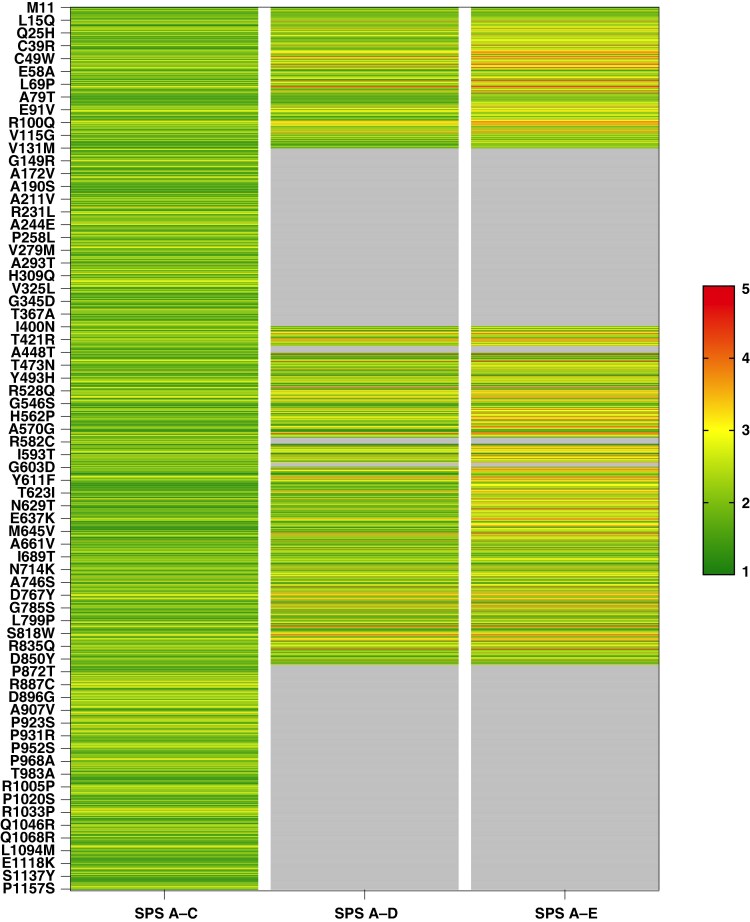
Structural scoring of all known *KCNH2* missense variants as distributed along the structural domains of hERG. The structural scoring predicted by our algorithm is provided in an ACMG-standardized manner (scoring from 1 to 5 in a continuous gradient – see colored bar) for structural Scores A to C (adapted to structural domains of hERG that lack CryoEM structural information), Scores A to D and Scores A to E (adapted to structurally characterized CryoEM regions). Grey zones are for non-structured domains. All scores are detailed in the Supplementary material online, *Table S1*.

The detailed procedure to reach the SPS and results for all missense variants are provided in Supplementary material online, *Table S1*. For each variant, we report: exon location, structural domain, nucleotide change, ClinVar and Cardiogen conditions, and ACMG annotations and classification. For structurally resolved domains, all five A to E scores are included; for unresolved regions (residues 132–398, 433–448, 511–519, 578–582, 598–602, and 864–1159, per CryoEM data), only SPS A–C are provided, with D and E scores being defaulted to zero. Among the missense variants, 827 (47.9%) fall within unresolved regions where D and E scores cannot be calculated, which by essence implies lower global scoring and pathogenicity discrimination. For the remainder, score E aggregates the number of interacting residues (up to 15 per variant), some of which are frequently mutated at known pathogenic positions – up to 12 in specific cases. These residues are listed in Supplementary material online, *Table S1*, offering a clear identification of structural hotspots that contribute to pathogenicity scoring. All five scores are normalized to a common 0–1 scale to ensure equal weighting in the final aggregate score used for variant prioritization and ACMG score correspondence. *Figure 3* summarizes the results of this multi-parametric structural scoring generated by our algorithm. SPS A–C are for non-structured domains, while SPS A–D and A–E are for structured hERG domains. SPS A–D and A–E are compared since E adds a probabilistic dimension to the global scoring, while individual scores A, B, C, and D illustrate severity. Several take-home messages can be taken: (i) SPS A–C can be in the high range in spite of lacking D and E scores, (ii) score D adds severity in scoring, and (iii) score E further adds pathogenicity where score D by itself is lacking discriminative power.

### The ACMG framework helps define a structural scoring threshold for pathogenic variant prediction

**Figure 4:**
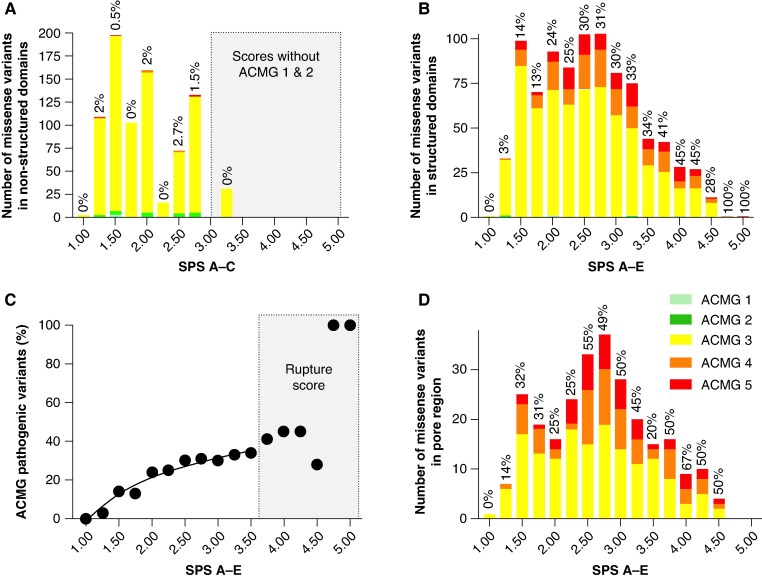
Distribution of variants by SPS and differentiated by ACMG classification using color coding. *A*, Representation of variants distribution for non-structured hERG domains and possessing scores A to C. The shaded grey area marks the SPS for which no ACMG class 1 or class 2 can be detected. The percentage shown above each bar indicates the proportion of pathogenic variants (class 4 and class 5). *B*, Representation of variants having the five different scores A to E within structured domains. *C*, Percentage of pathogenic ACMG variants as a function of the SPS value for all structured regions. The shaded grey area indicates a rupture point in the evolution of pathogenicity. *D*, same as *B* but limited to the pore region.

While the algorithm provides scores for all variants, we needed to establish the appropriate threshold for detecting pathogenicity. Thus, we calibrated the multi-parametric structural scoring by making use of the ACMG classification as an index (*Figure 4*). Since we wanted to test both scoring systems, we needed to calibrate both SPS A–C and A–E independently. Finally, we also calibrated SPS A–E independently for the pore region that is clearly a more sensitive structural domain to sequence variations than others (*Figure 2*). Several interesting observations can be made by looking at the distribution of ACMG scores as a function of SPS values. First, consistent with our first take-home message above, sequence variations in non-structured domains seldom lead to pathogenic ACMG classification, suggesting that these ion channel regions are highly tolerant to amino acid alterations (*Figure 4A*). Also, most of the benign variants are localized in non-structured regions, which is consistent with this high tolerance to sequence fluctuation (24 out of the 30 in total). If calibration of the SPS is performed with regard to the absence of ACMG class 1 & 2, then an SPS >3 is likely to indicate pathogenicity. Shifting to structured hERG domains clearly demonstrates that there is a correlation between the SPS and the proportion of ACMG pathogenic variants indicating that high scores lead to better pathogenicity predictions (*Figure 4B*). A fit of the percentage of ACMG class 4 & 5 as a function of the SPS indicates saturation at 3.25 (up to 34% of ACMG class 4 & 5 variants), which could represent an interesting threshold level for maximal probability of pathogenicity detection (*Figure 4C*). Above a threshold of 3.5, a rupture point can be detected defining a scoring domain in which the percentage of ACMG pathogenic variants becomes really high (up to 100% for scores 4.75 and 5; *Figure 4C*). Interestingly, the proportions of ACMG pathogenic variants are even higher along the scale of the SPS for the pore region, confirming the higher sensitivity of this domain to sequence variations (*Figure 4D*). In the pore region, an SPS of 2.5 means that at least 55% of the variants will lead to ACMG severity class 4 or 5, while if all structured regions are taken as a whole, this percentage drops to 30%.

In summary, SPARC provides lower SPS values in non-structured domains, and the scoring is consistent with an almost complete absence of ACMG class 4 & 5 variants in these domains. A pathogenicity threshold of 3.25 appears reasonable to predict variant pathogenicity in these non-structured domains due to the complete lack of ACMG class 1 or 2 variants at SPS value of 3.0 or above. In structured hERG domains, the SPS nicely correlate with the probability to have ACMG class 4 & 5 variants with maximal probabilities occurring at a threshold score of 3.25. Hence, a threshold SPS of 3.25 can be taken as a rather reliable index for maximal probability to detect pathogenicity for VUS (31 for non-structured hERG domains and 141 for structured domains). An additional 52 VUS of the pore region may be pathogenic if a lower SPS of 2.5 is taken into account.

### Detection of pathogenic variants using structure-independent Scores A to C

**Figure 5:**
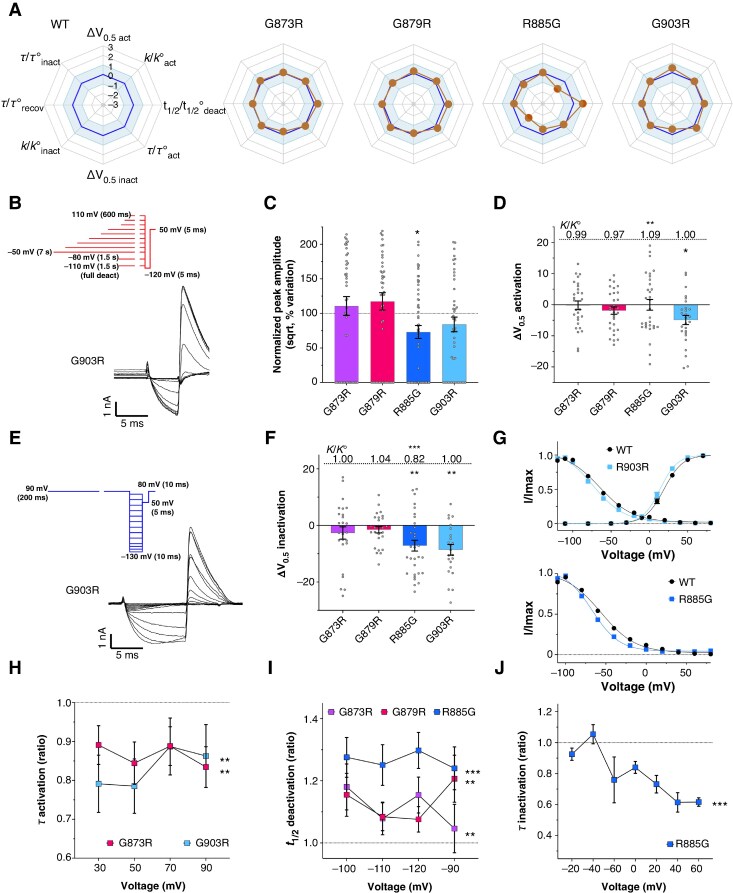
Functional characterization of variants predicted pathogenic with scores A to C. *A*, Radar plots summarizing the eight biophysical parameters of hERG besides current amplitude. The blue line corresponds to the wild-type mean situation, with light blue surface area representing the standard deviation of the wild-type condition. The orange dots correspond to variant normalized values in the homozygous condition. Loss-of-function deviation goes towards the center of the radar, while, conversely, gain-of-function is directed outward. *B*, Steady-state activation protocol to measure I_max_ and V_0.5_ of activation, along with a representative recording for the G^903^R variant. *C*, Variation of normalized root-square peak amplitude (sqrt) for four variants compared to wild-type condition. *D*, Variation of activation V_0.5_ for four variants compared to wild-type condition. The ratios of the slopes of voltage dependence of activation are given as values on top (K for the variant, K° for the wild-type). *E*, Steady-state inactivation protocol to measure V_0.5_ of inactivation, along with a representative recording for the G^903^R variant. *F*, Variation of inactivation V_0.5_ for four variants compared to the wild-type condition. Slope ratios of the voltage dependence of inactivation are given as values on top. *G*, Superimposed activation and inactivation curves for wild-type and G^903^R, and superimposed inactivation curves for wild-type and R^885^G. *H*, Activation time constants of two variants relative to wild-type. *I*, Half-deactivation time of three variants relative to wild-type. *J*, Inactivation time constant of R^885^G relative to wild-type. The experiments were performed in two technical replicates. The *n*-values were between 47 and 69 for the amplitude (*C*) and between 10 and 31 for the biophysical parameters (*D*,*F–J*). For details, see Supplementary material online, *Table S1*. Statistical analyses were performed using parametric t-tests for amplitudes and biophysical parameters, and ANOVA for hERG channel kinetic parameters measured at multiple voltages. Significance levels are indicated as follows: *P < 0.05; **P < 0.01; ***P < 0.001.

The combination of the three structure-independent scores (A, B, and C), adapted to the ACMG classification framework, is by itself capable of identifying several potentially pathogenic hERG variants. Specifically, this approach detected 52 variants with a SPS A–C ≥ 3.25, suggesting likely pathogenicity. 31 of these missense variants are exclusively located in non-structured hERG domains (10 from the N-linker and 21 from the C-tail – all with SPS A–C = 3.25; Supplementary material online, *Table S1*, *Figure 3*). They are all VUS according to the ACMG classification. Remarkably, this A to C scoring approach consistently highlights glycine-to-arginine (G->R) and arginine-to-glycine (R->G) substitutions as generally pathogenic (18 G->R and 13 R->G substitutions). Interestingly, this holds true also for the remaining 21 variants that are also scored on D and E parameters and that reach this 3.25 SPS A–C threshold or more (16 G->R and 5 R->G substitutions – 6 of them being ACMG class 4 or 5 and the 15 remaining ones being VUS; Supplementary material online, *Table S1*). Next, to determine whether a SPS of 3.25 in non-resolved hERG structures could lead to prediction of functional defects, we characterized four VUS from the Bamacoeur database (G^873^R, G^879^R, R^885^G and G^903^R), using a streamlined functional assessment protocol^16^ adapted for automated patch-clamp electrophysiology (see Supplementary material online, *Figure S8*). Their biophysical profiles are summarized *via* radar plots, using wild-type hERG as reference point, along with its standard deviation (*Figure 5A*). Variants G^873^R and G^879^R showed features consistent with a mild gain-of-function phenotype: no significant change in current amplitude (*Figure* *5B* and *C*), faster activation for G^879^R (*Figure 5H*), and slower deactivation for both variants (*Figure 5I*). The other two C-tail variants, R^885^G and G^903^R, displayed mixed functional effects. Loss-of-function features included reduced current amplitude for R^885^G (∼25% decrease; *Figure 5C*), significant changes in the slopes of voltage dependence of activation and inactivation for R^885^G, a leftward shift in the voltage dependence of inactivation for both (>5 mV; *Figure* *5F* and *G*), and accelerated inactivation for R^885^G (*Figure 5J*). Gain-of-function effects included slowed deactivation (R^885^G, *Figure 5I*), faster activation, and a leftward shift in activation for G^903^R (*Figure 5D, G*). These findings highlight the algorithm’s potential to detect both gain- and loss-of-function variants and underscore the importance of evaluating a comprehensive set of electrophysiological parameters, beyond current amplitude alone. Thus, we conclude that this A–C structural scoring approach can flag variants with subtle functional effects. These subtle effects are consistent with the fact that these non-structured regions can more easily accommodate sequence variations than structured regions as witnessed by the low percentage of variants classified as class 4 or 5 according to ACMG standards (*Figure 4A*).

### Value of a full A-E SPS

SPARC is meant to be a binary classifier. Variants above a certain SPS threshold should exhibit a high pathogenic risk on the basis of significant alterations of hERG channel biophysics assessed by patch-clamp. We tested if the SPS threshold of 3.25 is high enough to minimize false positives (FP). Accordingly, SPS values below the threshold define variants of uncertain pathogenic risk and should not be interpreted as evidence of a benign nature. Also, SPARC should be solid enough that variants classified as benign both clinically (ACMG class 1 or 2) and further confirmed functionally (based on patch-clamp experiments) should be predicted as non-pathogenic. We assessed the validity of these claims.

**Figure 6:**
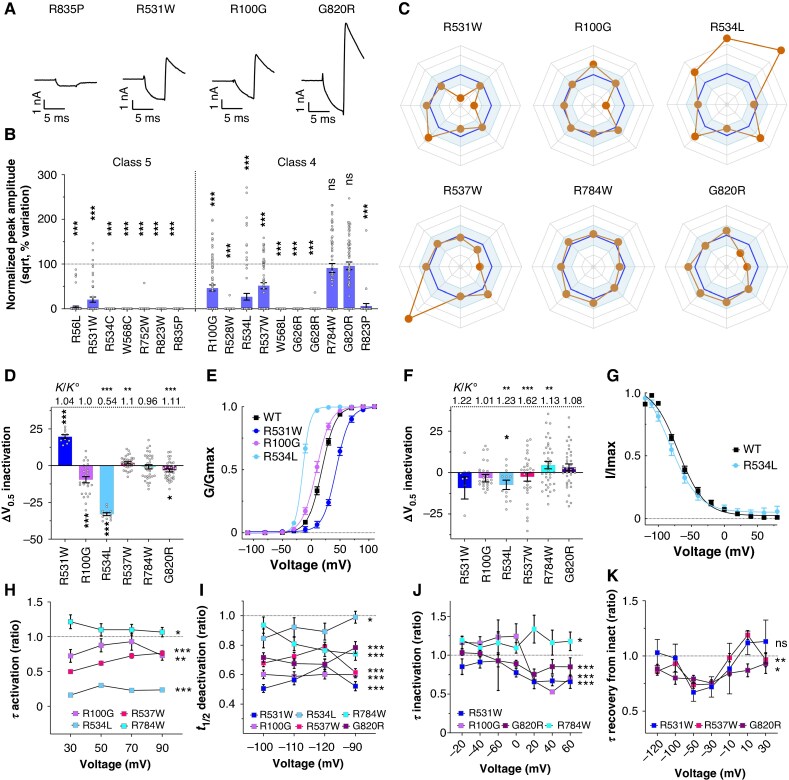
SPARC scoring on classes 4 and 5 variants. *A*, Representative current recordings of two class 5 variants (R^835^P and R^531^W) and two class 4 variants (R^100^G and G^820^R). *B*, Variation of normalized peak amplitude (sqrt) compared to wild-type condition for seven class 5 and ten class 4 variants. *C*, Radar plots summarizing the variations in the eight additional biophysical parameters. Axes are similar to *Figure 5A*. *D*, Variation of activation V_0.5_ for six variants compared to wild-type condition and associated k/k° values of activation. *E*, Superimposed activation curves for wild-type and three variants. *F*, Variation of inactivation V_0.5_ for six variants compared to wild-type condition and associated k/k° values of inactivation. *G*, Superimposed inactivation curves for wild-type and R^534^L. *H*, Activation time constants of four variants relative to wild-type. *I*, Half-deactivation time of six variants relative to wild-type. *J*, Inactivation time constant of four variants relative to wild-type. *K*, Time constant of recovery from inactivation for three variants relative to wild-type. The experiments were performed in two technical replicates. The *n*-values were between 26 and 132 for the amplitude (*B*) and between 5 and 37 for the biophysical parameters (*D–K*). For details, see Supplementary material online, *Table S1*. Statistical analyses were performed using parametric t-tests for amplitudes and biophysical parameters, and ANOVA for hERG channel kinetic parameters measured at multiple voltages. Significance levels are indicated as follows: *P < 0.05; **P < 0.01; ***P < 0.001. All recordings in homozygous conditions.

**Figure 7:**
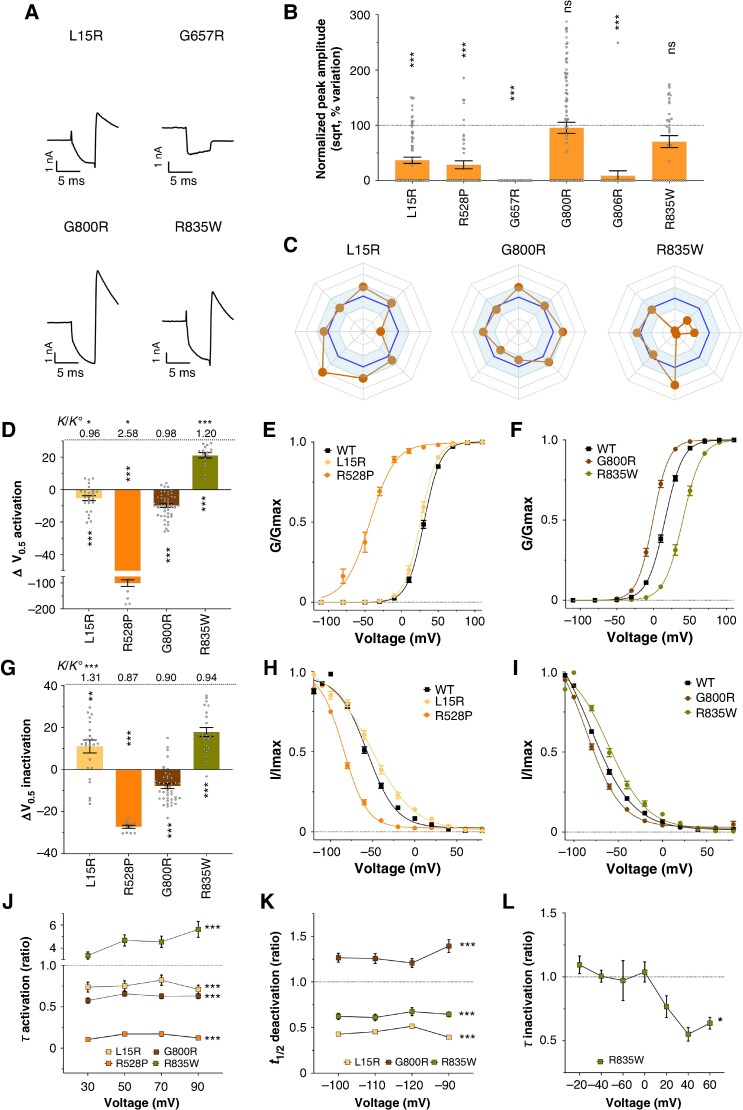
SPARC scoring on VUS. *A*, Representative current recordings of four class 3 variants. *B*, Variation of normalized peak amplitude (sqrt) compared to wild-type condition for six class 3 variants. *C*, Radar plots summarizing the main biophysical parameters. Axes are similar to *Figure 5A*. *D*, Variation of activation V_0.5_ for four variants compared to wild-type condition. Top values: ratios of slopes. *E*,*F* Superimposed activation curves for wild-type and four variants. *G*, Variation of inactivation V_0.5_ for four variants compared to wild-type condition. Top values: ratios of slopes. *H,I*, Superimposed inactivation curves for wild-type and four variants. *J*, Activation time constants of four variants relative to wild-type. *K*, Half-deactivation time of three variants relative to wild-type. *L*, Inactivation time constant of R^835^W relative to wild-type. The experiments were performed in two technical replicates. The *n*-values were between 21 and 99 for the amplitude (*B*) and between 9 and 49 for the biophysical parameters (*D–I*). For details, see Supplementary material online, *Table S1*. Statistical analyses were performed using parametric t-tests for amplitudes and biophysical parameters, and ANOVA for hERG channel kinetic parameters measured at multiple voltages. Significance levels are indicated as follows: *P < 0.05; **P < 0.01; ***P < 0.001. Recordings in homozygous conditions.

**Figure 8:**
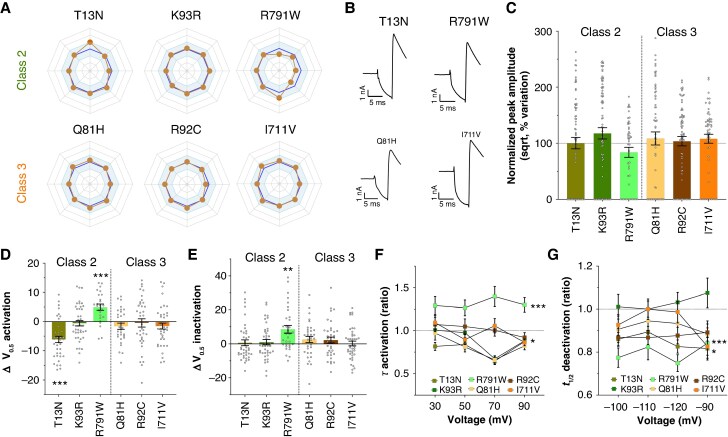
Functionally benign variants from ACMG classes 2 & 3 confirmed to be benign by SPARC. *A*, Radar plots summarizing the main biophysical parameters. Axes are similar to *Figure 5A*. *B*, Representative current recordings of four variants (two upper from class 2 and two lower from class 3). *C*, Variation of normalized peak amplitude compared to wild-type condition for six variants. *D*, Variation of activation V_0.5_ for six variants compared to wild-type condition. *E*, Variation of inactivation V_0.5_ for six variants compared to wild-type condition. The ratio *k*/*k*° of activation and inactivation did not differ significantly from WT across all variants. *F*, Activation time constants of six variants relative to wild-type. *G*, Half-deactivation time of six variants relative to wild-type. The experiments were performed in two technical replicates. The *n*-values were between 46 and 77 for the amplitude (*C*) and between 12 and 45 for the biophysical parameters (*D–G*). For details, see Supplementary material online, *Table S1*. Statistical analyses were performed using parametric t-tests for amplitudes and biophysical parameters, and ANOVA for hERG channel kinetic parameters measured at multiple voltages. Significance levels are indicated as follows: *P < 0.05; **P < 0.01; ***P < 0.001. Recordings in homozygous conditions.

To evaluate the strength of SPARC, we examined the 229 variants identified as of high pathogenic risk by the algorithm using the complete five-score system (A to E, SPS ≥3.25 on the basis of structured hERG domains). Of these, 86 were also classified as pathogenic by ACMG (38 as class 5 and 48 as class 4). The remaining 143 consisted of 141 class 3 (VUS) variants, 1 class 2 variant and 1 class 1 variant. This initial result supports the reliability of SPARC, with 38% of high-scoring variants confirmed as pathogenic by ACMG (see Supplementary material online, *Table S1*, *Figure 4B*). To further validate these predictions, for reasons of workload, we randomly selected 24 variants from the Bamacoeur database out of the 229 algorithm-identified ones (see Supplementary material online, *Figure S9*) and generated them *via* site-directed mutagenesis in the hERG-expressing plasmid. These were then subjected to full biophysical analyses using our established fast-track patch-clamp protocol.^16^ The selected variants included 7 ACMG class 5 (R^56^L, R^531^W, R^534^C, W^568^C, R^752^W, R^823^W, R^835^P) (*Figure 6*), 10 class 4 (R^100^G, R^528^W, R^534^L, R^537^W, W^568^L, G^626^R, G^628^R, R^784^W, G^820^R, R^823^P) (*Figure 6*) and 6 class 3 (L^15^R, R^528^P, G^657^R, G^800^R, G^806^R, R^835^W) (*Figure 7*) and 1 class 2 variant (R^791^W) (*Figure 8*). Among these, 19 variants caused a reduction in current amplitude of more than 60%, and 13 produced no measurable current at all, preventing further analyses (*Figures 6B, 7B*; Supplementary material online, *Table S1*). These included 6 class 5 variants (R^56^L, R^534^C, W^568^C, R^752^W, R^823^W, R^835^P), 5 class 4 variants (R^528^W, W^568^L, G^626^R, G^628^R, R^823^P), and 2 class 3 variants (G^657^R, G^806^R). This finding suggests that at least two of the class 3 VUS could be reclassified as severely pathogenic based on their functional phenotype (*Figure 7B*). Additionally, 15 of the 17 ACMG class 4–5 variants were confirmed to be pathogenic based on this primary criterion of amplitude (*Figure 6B*). For the 10 variants with measurable current (6 ACMG class 4 or 5, and 4 VUS), we conducted detailed electrophysiological analyses (*Figures 6 & 7*; Supplementary material online, *Table S1*). We first verified the pathogenic profiles of the remaining class 5 (R^531^W) and class 4 variants (R^100^G, R^534^L, R^537^W, R^784^W, G^820^R). R^531^W showed a severe loss-of-function phenotype, with a 74% reduction in current amplitude (*Figure 6A,B*), a + 20-mV shift in voltage-dependent activation (*Figure 6D,E*), faster deactivation (*Figure 6I*), accelerated inactivation (*Figure 6J*), and faster recovery from inactivation (*Figure 6K*) – the latter being a gain-of-function effect (as monitored at three potentials, −50, −30 and −10 mV). This example illustrates the importance of covering all hERG channel properties to meaningfully interpret the causes of variant pathogenicity. Concerning class 4 variants, R^100^G also exhibited significant alterations: a 53% reduction in current amplitude (*Figure 6A,B*), nearly twofold faster deactivation (*Figure 6I*), a leftward shift in activation voltage (*Figure 6D,E*), and faster activation kinetics (*Figure 6H*), indicating both loss- and gain-of-function components. R^534^L showed a similarly complex phenotype (*Figure 6B*), with a 70% current amplitude loss (*Figure 6B*), a 7-mV leftward shift in inactivation (*Figure 6F,G*), and accelerated deactivation (*Figure 6I*). Gain-of-function traits included a substantial 33-mV leftward shift in activation and faster activation kinetics, along with significant changes in the slopes of voltage dependence activation and inactivation (*Figure 6H*). R^537^W also displayed a mixed phenotype, with a 45% reduction in current (*Figure 6B*), faster deactivation (*Figure 6I*), and enhanced activation and recovery from inactivation (*Figure 6H,K*). Both slopes of voltage dependence of activation and inactivation were affected (large change for inactivation). R^784^W had the mildest phenotype, presenting only subtle changes, including slightly faster deactivation (*Figure 6I*), slower inactivation (*Figure 6J*), and altered slope of voltage-dependence of inactivation (*Figure 6F*). Lastly, the G^820^R variant exhibits a mixed functional phenotype (*Figure 6C*). It shows loss-of-function effects through accelerated deactivation (*Figure 6I*) and slope of voltage-dependence of activation (*Figure 6D*). Also, a gain-of-function effect occurs through faster recovery from inactivation (*Figure 6K*). Notably, if the pathogenicity of the R^784^W and G^820^R variants had been assessed solely based on current amplitude, their disease potential would have been overlooked. This underscores the importance of evaluating the full spectrum of biophysical properties, as pathogenicity can arise from complex profiles involving both loss- and gain-of-function mechanisms.

Finally, we examined the biophysical properties of the four remaining VUS – L^15^R, R^528^P, G^800^R, and R^835^W – that exhibited sufficient potassium currents for detailed characterization. The L^15^R variant showed a severe mixed phenotype (*Figure 7C*), with a marked loss-of-function reflected by a 64% reduction in current amplitude (*Figure 7A,B*) and a twofold increase in deactivation rate (*Figure 7K*). At the same time, gain-of-function effects were observed across six parameters: a leftward shift in the voltage-dependence of activation (*Figure 7D,E*) along a change in slope, faster activation kinetics (*Figure 7J*), an 11-mV rightward shift in the voltage-dependence of inactivation, also along a change in slope (*Figure 7G,H*), and accelerated recovery from inactivation (*Figure 7C*). The R^528^P variant displayed a substantial reduction in current (*Figure 7B*), limiting the assessment of some biophysical parameters. However, it demonstrated loss-of-function *via* a pronounced leftward shift in the voltage-dependence of inactivation (27 mV) (*Figure 7G,H*), and gain-of-function through an extreme leftward shift in activation (101 mV), accompanied by an important change in slope (*Figure 7D,E*) as well as faster activation kinetics (*Figure 7J*). G^800^R also presents a mixed loss- and gain-of-function profile (*Figure 7C*). Loss-of-function is witnessed mainly by a negative shift in voltage-dependence of inactivation (*Figure 7G,I*). Gain-of-function is characterized by a leftward shift in voltage-dependence of activation (*Figure 7D,F*), accelerated activation (*Figure 7J*), and slowed deactivation (*Figure 7K*). Among all variants, R^835^W exhibited the most striking and pathogenic phenotype despite only a mild reduction in current amplitude (*Figure 7A,B*). This variant combined several loss-of-function traits – including a rightward shift in activation voltage-dependence, together with a major change in slope (*Figure 7D,F*), delayed activation (*Figure 7J*), and faster deactivation (*Figure 7K*) – with gain-of-function features such as an 18-mV rightward shift in the voltage dependence of inactivation (*Figure 7G,I*) and faster recovery from inactivation (*Figure 7C*). These findings demonstrate that all VUS predicted as pathogenic by SPARC indeed showed pathogenic biophysical profiles upon experimental validation. This strongly supports the utility of SPARC in guiding clinical interpretation of VUS, especially for clinicians without access to laboratory facilities. Additionally, SPARC reliably confirmed the presumed pathogenicity of ACMG class 4 and class 5 variants.

To test the next claim, we examined variants classified as benign according to ACMG criteria (classes 1 and 2). Among the 1727 hERG missense variants analyzed, only 30 fell into these benign categories (see Supplementary material online, *Table S1*). SPARC scoring largely supported this classification, with the notable exception of variant R^791^W, which received a SPS of 3.25 (*Figure 4B*). Interestingly, only 6 of the 30 benign variants were located in structurally characterized hERG regions, where all five scoring criteria (**A** to **E**) could be applied – T^13^N, K^93^R, L^109^R, V^491^I, V^533^G, and R^791^W. Given the borderline score of 3.25 for R^791^W, we performed a functional analysis to clarify its impact (*Figure 8*). R^791^W exhibited a normal current amplitude (*Figure* *8B* and *C*), but also showed a combination of opposing functional effects: a loss-of-function *via* a rightward shift in the voltage-dependence of activation (5 mV), slower activation kinetics (*Figure 8F*), and faster deactivation (*Figure 8G*), alongside a gain-of-function *via* a rightward shift in the voltage-dependence of inactivation (8.5 mV) (*Figure 8F*). Although these effects are significant, their opposing nature may balance out, leading to an overall benign phenotype. We also evaluated the biophysical behavior of two additional ACMG class 2 variants – T^13^N and K^93^R – that had low SPS value (2.5 and 1.25, respectively). Both exhibited functional profiles very similar to wild-type hERG (*Figure 8*). T^13^N showed only minor changes, with a modest gain-of-function in voltage-dependence of activation and activation kinetics, and a compensatory loss-of-function *via* faster deactivation (*Figure 8D,F,G*). K^93^R was virtually indistinguishable from wild-type across all parameters.

To further test SPARC, we analyzed three VUS variants – Q^81^H, R^92^C, and I^711^V – that appeared functionally benign (*Figure 8*). Consistent with expectations, these variants also had low SPS values: I^711^V scored 1.25, Q^81^H scored 2, and R^92^C scored 2.75, all <3.25 threshold (see Supplementary material online, *Table S1*). These findings support the reliability of SPARC in confirming truly benign variants when the functional analysis says so. Nonetheless, broader validation across a larger set of VUS is needed to solidify these preliminary conclusions.

## Discussion

### Usefulness of SPARC, a structure-based algorithm

ClinVar currently reports 3658 hERG gene variants, of which 1406 are classified as VUS – a number that continues to grow each month. Despite advances in high-throughput technologies, functionally characterizing such a large volume of variants remains an enormous task.^14^ In a recent review, we emphasized the urgent need for a comprehensive and standardized approach to assessing ion channel function, highlighting the valuable role that *in silico* methods can play in this context.^5^ The present project was inspired by our initial analysis of a smaller set of variants, in which we observed that those predicted to be pathogenic based on structural criteria were indeed functionally pathogenic.^16^ Building on that foundation, we extended our structural analysis to include almost all hERG missense variants (1727 out of 1752 at the date of submission) reported in ClinVar and in Bamacoeur, a French repository database. In parallel, we refined the original algorithm used to assess structural impact by introducing several key improvements: (i) expanding from 3 to 5 structural scores, (ii) applying equal weighting to each score, (iii) integrating known pathogenic hotspots into the scoring system, and (iv) calibrating the total structural score from 1 to 5 to indicate severity of hERG channel structural alterations. We developed SPARC, a Python-based tool with a user-friendly interface to automate this structural analysis, which we are making freely available to the scientific and medical community, and that can be adapted and used for other ion channel types as well. The usefulness of SPARC, a binary classifier, was validated by functional data on a series of 33 variants using a standardized, fast-track automated patch-clamp protocol. The conclusions are as follows: (1) an SPS threshold of 3.25 emerged as a good but possibly perfectible cut-off for detecting variants' pathogenicity, and (2) benign variants, as classified by ACMG or functional analyses, systematically displayed SPS <3.25. A drawback, however, is that the contrary is not true: SPS <3.25 is not systematically indicative of a lack of pathogenicity by ACMG or functional standards. Consequently, our scoring should not be directly correlated with the ACMG score. In a way, this is a strength, since available ACMG data on pathogenicity are highly imbalanced, with confirmed pathogenic cases vastly outnumbering benign ones. For example, treating only classes 1 and 5 as robust (0.46% and 5.73%, respectively), the data gives us 93% pathogenicity, making sensitivity a very difficult goal, possibly explaining the questionable results of other statistical/machine-learning classifiers. Also, one should be careful not to over-interpret the value of SPS, as it may not strictly indicate a pathogenicity severity. High scores for variants located within the ion conduction pore of the channel will obviously not predict the same functional severity as high scores for variants located in other channel regions. It should be mentioned also that functional severity restricted to hERG amplitude defects is difficult to translate into clinical terms. Indeed, our extensive functional analyses of hERG variants indicate that pathogenicity may arise from a wide range of alterations across various biophysical properties, sometimes involving intricate combinations of loss- and gain-of-function effects. These complexities highlight the need for new analytical strategies to fully interpret variant impact. Nevertheless, in spite of these difficulties, this approach helped reclassify one variant (R^791^W) originally labeled benign by ACMG standards that showed both structural and functional evidence of pathogenicity.

### Benchmarking SPARC with AlphaMissense and REVEL

We have performed a systematic comparison of our scores for each individual variant with scores obtained from AlphaMissense^11^ and REVEL.^12^ Unfortunately, REVEL data are sparse (total of 114 missense variants analyzed; 6.6%). Both algorithms appear to largely overcall pathogenicity. This is particularly noticeable in the case of REVEL. On CryoEM-resolved structures of hERG, AlphaMissense identifies as ‘Likely-pathogenic’ 47.9% of the variants, while REVEL does it for 100% of the variants. Interestingly, what REVEL classifies as ‘Likely-pathogenic’ is systematically ‘Ambiguous’ for AlphaMissense. These simple considerations question the validity of these approaches in detecting pathogenicity. If one now considers non-resolved CryoEM structures, wherein we detected very little pathogenicity, AlphaMissense considers as ‘Likely-pathogenic’ 11.4% of the variants, while 80.4% would be ‘Likely-benign’. This eventually makes sense. However, for REVEL, 100% of the variants (65 out of 114 analyzed) would be ‘Likely-pathogenic’ which tends to indicate that REVEL systematically considers as pathogenic each new variant. It therefore has no discriminative power at all. We next benchmarked our algorithm by comparing it to AlphaMissense and REVEL (see Supplementary material online, *Table S1*). We did this on the scores ≥3.25 which we trust from our algorithm to detect pathogenicity without indication of severity. The comparison is of interest: 215 out of 260 of our pathogenic variants were also pathogenic with AlphaMissense (82.7%) and, when available, with REVEL (100%, 9 variants in common only). However, our algorithm detected 45 additional variants that were not detected by AlphaMissense (classified as ‘likely benign’ (36 variants) or ‘Ambiguous’ (9 variants)). Of these undetected variants, we have tested 5 variants that all show significant alterations in biophysical properties compared to wild-type (R^100^G, G^873^R, G^879^R, R^885^G, G^903^R). These data demonstrate two points: (i) our algorithm does not overcall pathogenicity contrary to REVEL, and (ii) we detect variants with functional differences compared to wild-type with better definition than AlphaMissense.

### Limitation of SPARC

SPARC has some limitations. One major constraint is that certain regions of the hERG channel – particularly flexible intracellular domains such as the N-linker and C-terminus – lack structural data. This feature limits scoring to only three criteria (A–C) in those areas. Nevertheless, we observed that some variants in these unstructured regions still received scores >3.25 and demonstrated functional pathogenicity, though the scoring accuracy was clearly enhanced when all five criteria could be applied. This underscores the need for a more complete hERG structural model. However, as we noted that ACMG class 4 and 5 variants are extremely rarely associated to these regions, it indicates that sequence variations in these domains have a low probability to trigger pathogenicity. A second limitation is that our current scoring system may underrate pathogenicity in some structural regions, as mentioned earlier for the ion conduction pore. This suggests that a domain-specific adjustment to the pathogenicity threshold should be evaluated functionally with a larger dataset of variants. A third limitation includes the algorithm’s focus on local effects of amino acid substitutions, without modelling broader allosteric changes to the protein. Incorporating molecular dynamics simulations could help address this issue. Furthermore, the model currently performs in homozygous conditions and can hardly interpret what would happen in a heterozygous condition at the functional level. This remains true for all the bioinformatic tools developed so far. Also, it does not account for the influence of accessory proteins. It is likely that future CryoEM studies will reveal the full structure of the hERG protein complex, including interactions with partners as shown for ankyrin complexes.^23^ Amino acid changes that disrupt these interfaces may introduce new steric clashes not evident from the hERG structure alone, but which would elevate the structural pathogenicity of certain variants.

In conclusion, SPARC represents a valuable tool for rapidly assessing the pathogenic potential of novel hERG variants. When the structural score exceeds the 3.25 threshold, functional validation may not be necessary – offering clinicians a faster, less resource-intensive path to variant classification and, ultimately, diagnosis.

## Supplementary Material

euaf327_Supplementary_Data

## Data Availability

The Python macro of SPARC is available at https://doi.org/10.5281/zenodo.15422598, and the individual sheets for the scoring of hERG variants are at https://doi.org/10.5281/zenodo.15422598. All other data are summarized in the Supplementary material online, *Table S1*. Additional electrophysiological data (raw traces) underlying this article will be shared on reasonable request to the corresponding author.
