# Supplementary material for: SPARC: a structural pathogenicity algorithm for risk classification of hERG variants
Source: Europace. 2025 Dec 25;28(2):euaf327. doi: 10.1093/europace/euaf327 (PMC12877647; doi:10.1093/europace/euaf327)
Supplement: euaf327_Supplementary_Data [file euaf327_supplementary_data.zip › Suppl-Patho-Predict-Europace-V4.docx]

**SPARC : a Structural Pathogenicity Algorithm for Risk Classification of hERG Variants**

**Frank C. Chatelain^1,2,*^, Barbara Ribeiro de Oliveira^2,3,*^, Guillaume Grataloup^1^, Noé Robert^1^, Malak Alameh^2,3^, Aurélie Thollet^4^, Jérôme Montnach^2,3^, Sylvain Feliciangeli^1,2^, Aline Rio**^3^**, Floriane Bibault^3^, Delphine Bichet^1^, Olivier Bignucolo^1^, Fabrice Extramiana^5^, Rupamanjari Majumder^3^, Jean-Jacques Schott**^3^**, Vincent Probst^4^, Isabelle Denjoy^5^, Florian Lesage^1,2^, Gildas Loussouarn^2,3^, Michel De Waard^2,3,¶^**

*See excel file*

**Table S1**: Exhaustive list of the 1727 missense variants from ClinVar and the Bamacoeur database containing the relevant clinical and structural scoring details provided by the algorithm. The functional evaluation of the selected variants is provided. Functional parameters that were evaluated are: (i) percentage of variation of maximal current amplitude compared to wild-type hERG, (ii) steady-state activation parameters (V_0.5-act_, slope k_act_), (iii) activation gate kinetics (τ_act_, t_0.5-deact_), (iv) steady-state inactivation parameters (V_0.5-inact_, slope k_inact_) and (v) inactivation gate kinetics (τ_inact_, τ_recov_). The Alpha Missense and REVEL pathogenicity scoring and prediction are presented in addition to the SPS for each variant.

**Supplementary methods**

*SPARC scoring system*

*Improvements as compared to a previous report*

In a previous report^3^, we employed a systematic approach in which each variant was analyzed individually. For every amino acid substitution, three independent scores were assigned - reflecting changes in size, hydrophobicity, and charge - regardless of their structural impact. The mutation’s effect on the local environment of the substituted residue and on the overall channel structure was then evaluated using a three‑tier severity scale: no impact, compensatory impact, or non‑compensatory impact. This method, however, presented two major drawbacks: limited statistical robustness, which sometimes hindered precise determination of the final score, and a labor‑intensive workflow. Indeed, the process of measuring distances before and after substitution, sequentially analyzing steric clashes, and performing structural minimization calculations meant that only three to four variants could be examined per day.

The automated version introduced here retains the strengths of the previous approach - namely the scoring of size, hydrophobicity, and charge, together with structural analysis - while adding a statistical dimension that incorporates the probability of occurrence of different rotamers of neo‑residues. Moreover, this new method accounts for variability in the sensitivity of protein regions, including the presence of hot spots where mutations are more likely to affect channel function. Finally, automation ensures consistent objectivity across all variant analyses and removes the time constraints of manual implementation. With this software, we can now process several hundred variants per hour.

*Details of the scoring system*

**Score** **A**- Residue size alteration. For this criterion, we considered two parameters of major importance:

1. The accessible surface area of the amino acid side chains as described ^1, 2^ and
2. The Van der Waals volume determined by the nature of the side-chain of the amino acid ^4^.

**Table S2** illustrates the values reported for these two parameters for each amino acid in four former studies ^1, 2, 4, 5^.

**Table** **S2**. Standards of amino acid surface or volume. S_1_ and S_2_ come from 2 studies ^1, 2^ and define the accessible surface area used to assign score **A** to each substitution. V_1_ and V_2_ originate from 2 other studies that define the Van der Waals volumes to define score **A** also ^4, 5^.

| **Amino-acid** | **S_1_ (Å^2^)** | **S_2_ (Å^2^)** | **V_1_ (Å^3^)** | **V_2_ (Å^3^)** |
| --- | --- | --- | --- | --- |
| Ala | 67 | 44.1 | 67 | 88.6 |
| Arg | 196 | 152.9 | 148 | 173.4 |
| Asn | 113 | 80.8 | 86 | 114.1 |
| Asp | 106 | 76.3 | 91 | 111.1 |
| Cys | 104 | 56.4 | 86 | 108.5 |
| Gln | 144 | 100.6 | 114 | 143.8 |
| Glu | 138 | 99.2 | 109 | 138.4 |
| Gly | 0 | 0 | 48 | 60.1 |
| His | 151 | 98.2 | 118 | 153.2 |
| Ile | 140 | 90.9 | 124 | 166.7 |
| Leu | 137 | 92.8 | 124 | 166.7 |
| Lys | 167 | 139.1 | 135 | 168.6 |
| Met | 160 | 95.3 | 124 | 162.9 |
| Phe | 175 | 107.4 | 135 | 189.9 |
| Pro | 105 | 79.5 | 90 | 112.7 |
| Ser | 80 | 57.5 | 73 | 89 |
| Thr | 102 | 73.4 | 93 | 116.1 |
| Trp | 217 | 143.4 | 163 | 227.8 |
| Tyr | 187 | 119.1 | 141 | 193.6 |
| Val | 117 | 73 | 105 | 140 |

From these four sets of values, four matrices were built that provide, for each of the 20 possible natural amino acid substitutions, the absolute value of the difference in accessible surface area or in Van der Waals volume, depending on the study (S_1_, S_2_, V_1_ and V_2_) (**Figure S1**).
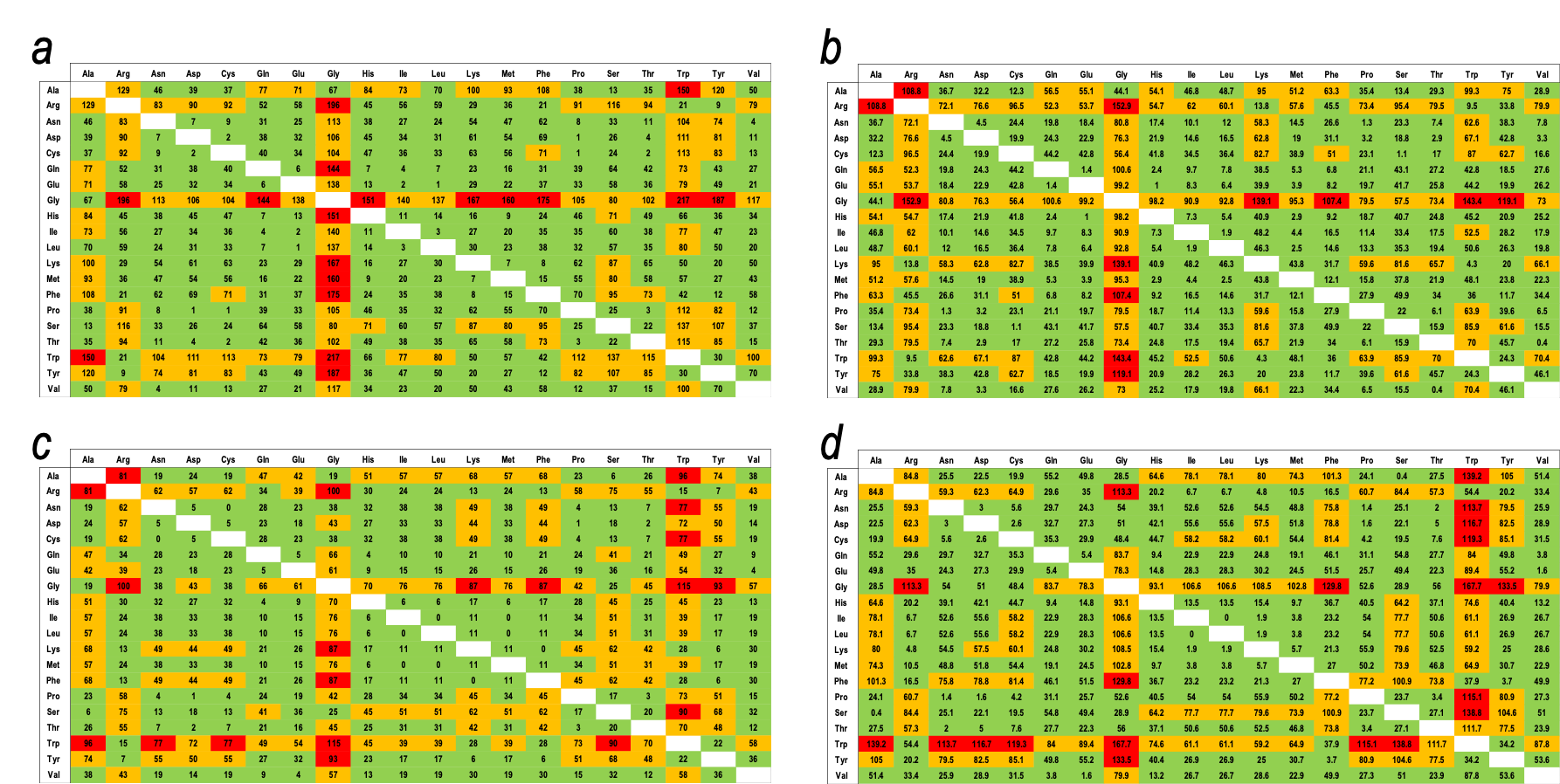


**Figure S1.** Size criteria matrices illustrating absolute value changes induced by amino acid substitutions. Matrices of the absolute values of accessible surface area (Å^2^) differences (***a***, S_1_, ***b***, S_2_, according to **Table S2**) and matrices of absolute values of Van der Waals volume (Å^3^) differences (***c***, V_1_, ***d***, V_2_, according to **Table S2**). Boxed values are colored according to the extent of maximal alteration: green from 0 to 33%, orange from 34 to 66% and red from 67 to 100%.

We then normalized to 1 all values to their maximum and built a second matrix for each of the four cases (S_1_, S_2_, V_1_ and V_2_). These new matrices describe **Score** **A** as a function of amino acid change (**Figure S2**).

**Figure S2.** Individual score **A** defined on the basis of surface and volume criteria matrices shown in **Figure S1**. ***a***, from S_1_ data. ***b***, from S_2_ data. ***c***, from V_1_ data. ***d***, from V_2_ data.

Finally, equal weight was given to the accessible surface area (S_1_ & S_2_) and the Van der Waals volume criteria (V_1_ & V_2_). This is allowed because of the quite linear correlation between surface and volume variations (as compared to S1, r^2^ = 0.91 for S2, 0.94 for V1 and 0.89 for V2). Therefore, a single median matrix was constructed that conveniently incorporates all the scores from these four matrices (**Figure S3**). This is the final matrix that will be used throughout the manuscript to assign **Score** **A** for the size criterion.


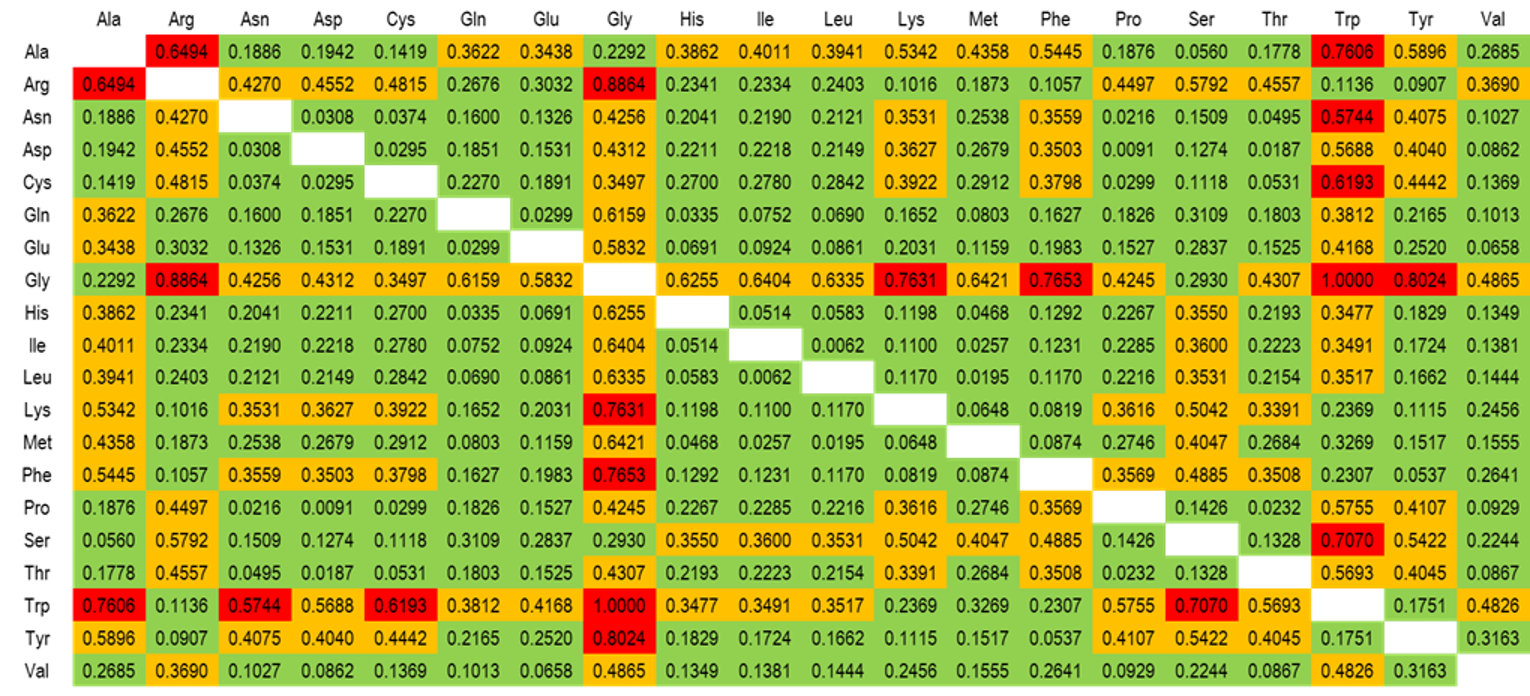


**Figure S3**. Median matrix for score **A** defined by the median value of the individual score **A** of the four matrices S_1_, S_2_, V_1_ and V_2_ described in **Figure S2**.

**Score** **B** - Hydrophobicity alteration. While Simm and collaborators report in a seminal review ^6^ the existence of 98 different hydrophobicity scales, **Score** **B** was defined along the recommendation lines of the UCSF Chimera software package. Four sources of information were used to establish the final hydrophobicity scoring matrix: i) the default hydrophobicity classification of amino acids according to the Kyte and Doolittle scale ^7^, which is the gold standard, and ii) three others assigning different hydrophobicity scores in different contexts (presence of a transmembrane context, a lipid bilayer, an α-helix or a β-sheet secondary structure) ^8-10^. **Table S3** illustrates in 4 columns from H_1_ to H_4_ the hydrophobicity standard values according to these 4 references.

**Table S3**. Standards of amino acid hydrophobicity based on UCSF Chimera software recommendations. H_1_: values from Kyte and Doolittle ^7^. H_2_: values from Hessa et al. ^8^. H_3_: values from Moon and Fleming ^9^. H_4_: values from Zhao and London ^10^. Note that unlike the H_1_ and H_4_ standards, a negative sign designates the most hydrophobic amino acids in the H_2_ and H_3_ standards.

Four hydrophobicity matrices were generated according to each standard (**Figure S4**).


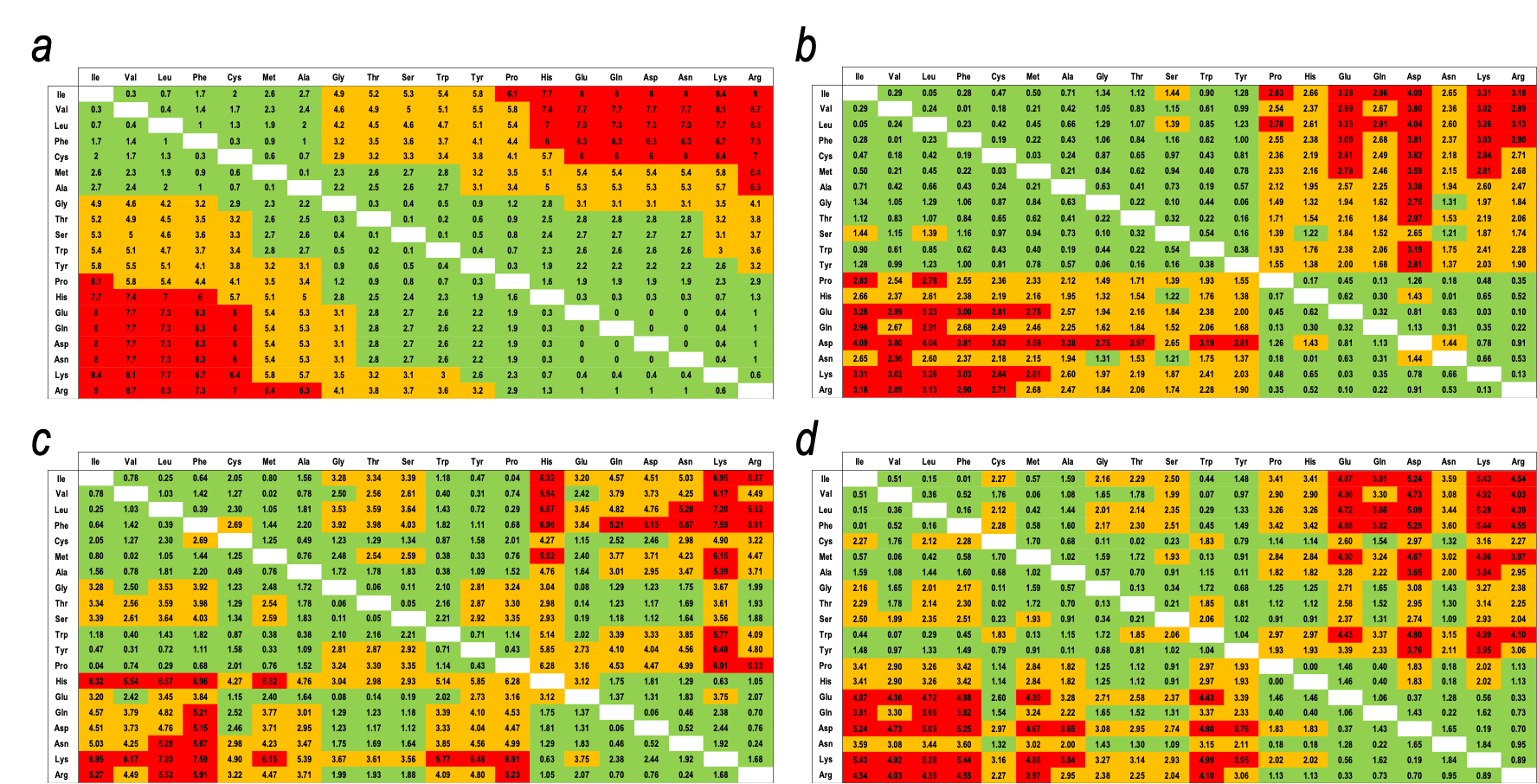


**Figure S4**. Hydrophobicity criteria matrices illustrating absolute value changes induced by amino acid substitutions. Matrices of the absolute values for hydrophobicity differences according to **Table S3**. ***a***-***d***: from H_1_ to H_4_. Boxed values are colored according to the extent of maximal alteration: green from 0 to 33%, orange from 34 to 66% and red from 67 to 100%.

Since parameters of interest are change in amplitudes and not direction of the change, only the absolute values of these variations were retained. Similarly, to **Score** **A**, each substitution was assigned a **Score** **B** evolving continuously from 0 to 1 according to the amplitude of this change. The resulting normalized matrices are shown in **Figure S5**.


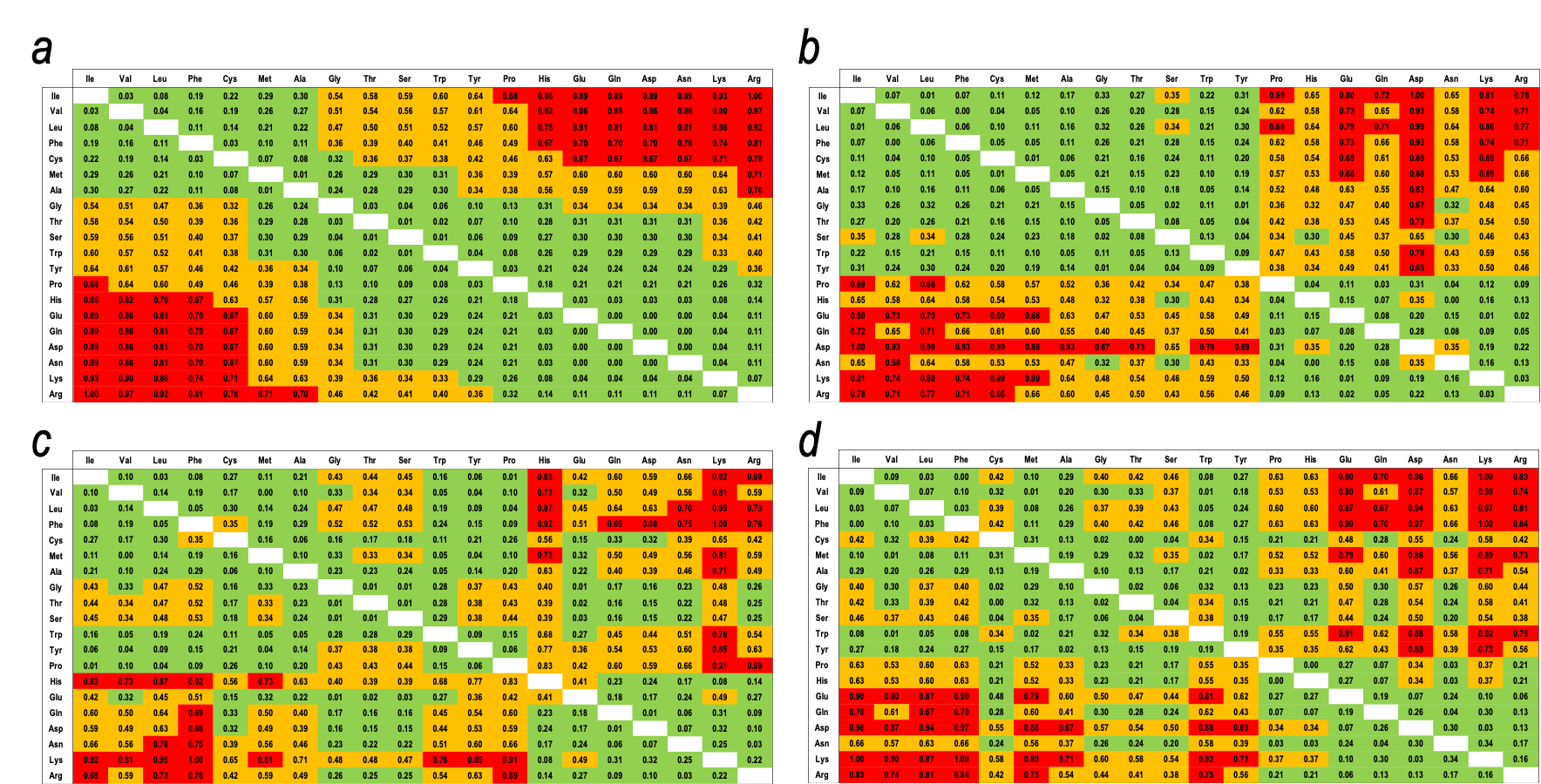


**Figure S5**. Individual score **B** defined on the basis of hydrophobicity criteria matrices shown in **Figure S4**. ***a-d***, from H_1_ to H_4_.

Finally, as we did for **Score** **A,** based on residue size criteria, a final **Score** **B** hydrophobicity matrix was built that represents the median values of these 4 matrices (**Figure S6**). This final matrix is used throughout the manuscript for the interpretation of variant impact on hERG structure.

**
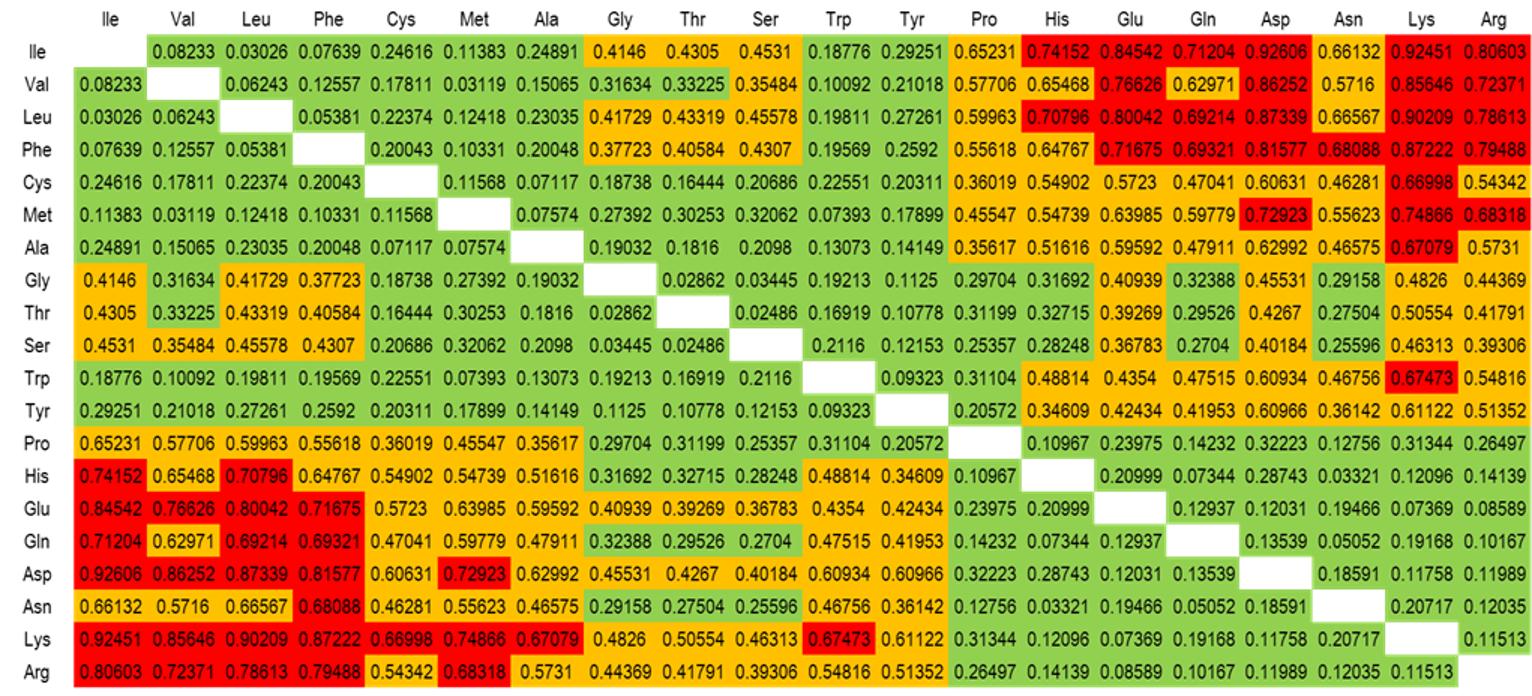
**

**Figure S6**. Median matrix for score **B** defined by the median value of the individual score **B** of the four matrices H_1_, H_2_, H_3_ and H_4_ described in **Figure S5**.

**Score** **C** – Charge alteration. The impact of an alteration in charge value induced by the amino acid substitution was scored independently of size (**Score** **A**) and hydrophobicity (**Score** **B**). An additional **Score** **C** was added that takes into account the changes in amino acid charge. Thus, **Score** **C** equals the charge difference and comes in discrete values (0 for no change, 0.5 for a single charge change, and 1 for the opposite charge introduction). Histidine may or may not exist in a charged form depending on the surrounding pH and the nature of the residues in its vicinity. Since the charge of histidine is difficult to predict, it was considered as charged throughout this study. **Figure S7** shows the corresponding matrix for **Score** **C**.

**
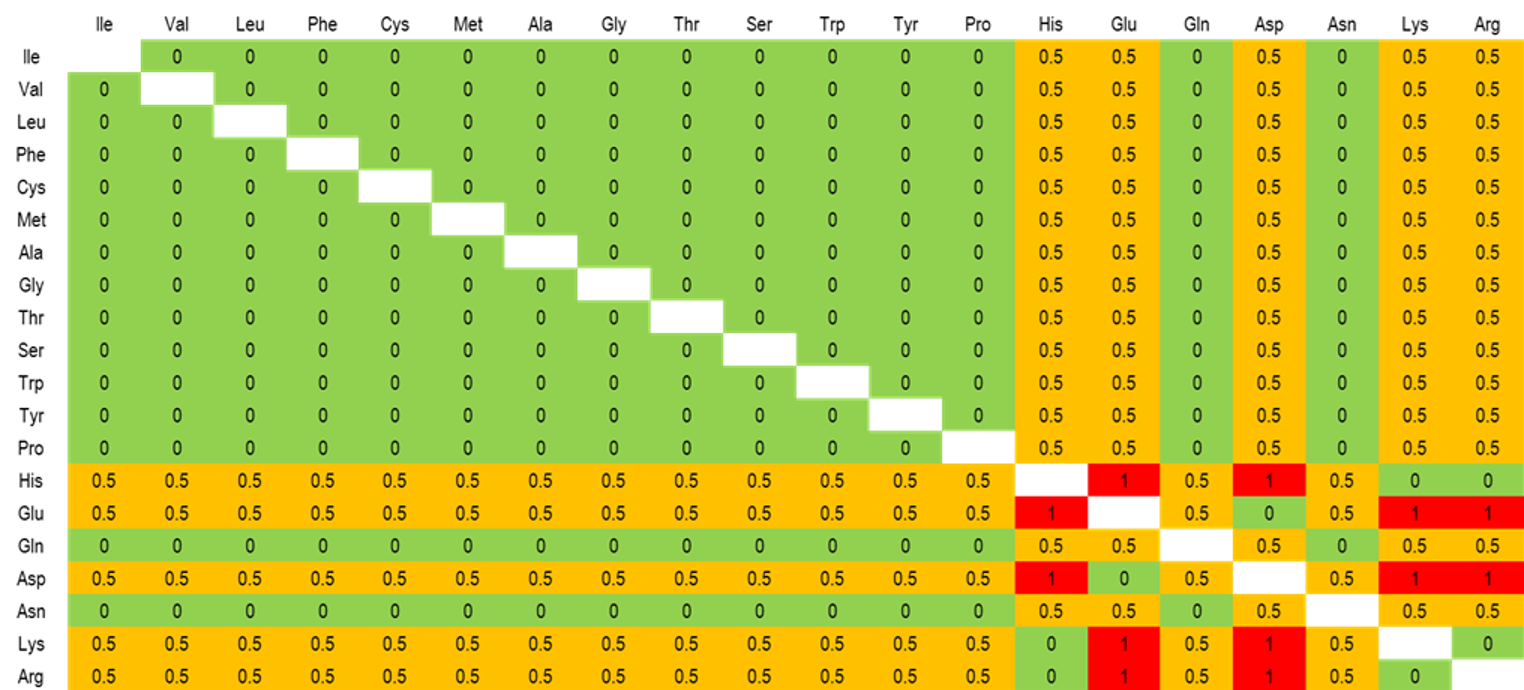
**

**Figure S7**. Matrix defining score **C** based on residue charge.

**Score** **D** - Clash severity. We used the "swapaa" function of UCSF Chimera to enable directed mutagenesis on a specific residue. This function draws from a variety of rotamer libraries, with each substitution presenting multiple rotamers defined by different side-chain orientations. For our analysis, we selected the Dunbrack library ^11^ to evaluate potential steric clashes induced by the various rotamers because it is the most commonly used. A steric clash occurs when two amino acids are so close together in a molecule that the electronic fields of the atoms that compose them overlap. Compensation being impossible, significant channel structural deformation will occur. The rotamers chosen for testing were those with a frequency of occurrence above 10% (up to 5 most frequent rotamers). If none occur with a frequency above 10%, then the three most frequent are tested. The "findclash" function was employed to identify clashes between the inserted rotamer and surrounding residues within a 5 Å radius. If no clash was detected, no further minimization was necessary, and a score of 0 (**Score** **D** = 0) was assigned to the analyzed rotamer. If one or more clashes were detected, hERG structure minimization was performed for each of the most probable rotamers of the newly introduced amino acid using the integrated minimization module of UCSF chimera. Accommodation could be achieved with less than 10 steps of minimization calculations. A score of 0.5 (**Score** **D** = 0.5) was assigned if the clashes were resolved through minimization, and a score of 1 (**Score D** = 1) was given if the clashes could not be accommodated. In the case of multiple rotamers for a given residue, the scores were weighted by the occurrence probability of each rotamer. For instance, if a variant yielded three main rotamers with occurrence probabilities of 10%, 30%, and 40%, respectively, and the associated scores were 0.5, 1, and 0, the final **Score** **D** would be calculated as (0.5×10 + 1×30 + 0×40) / 80 = 0.438. The maximal **Score** **D** is 1.

**Score** **E** – Pathogenic hotspot scoring. This score evaluates the potential pathogenicity of a variant based on the fraction of interacting amino acids that are known to be pathogenic when mutated. Within the hERG structure, a given variant is considered to pose a higher risk of pathogenicity if it directly interacts with one or more residues that have been reported as pathogenic in other contexts. To calculate this pathogenic hotspot score, we first identified the number of residues in contact with the variant for each of the most frequent rotamers (probability > 10%, up to five, as in **Score** **D**). For each selected rotamer: n is the number of neighboring residues in direct contact with the rotamer that are classified as pathogenic when mutated and N is the total number of residues in direct contact with the rotamer. **Score E** score is calculated as the sum, for each rotamer, of the product between the n/N ratio and its probability of occurrence: E = ∑ (n/N x Probability of occurrence). The maximal **Score** **E** is 1, consistent with other scoring systems. A **Score** **E** of 1 indicates that all rotamers interact with residues that are pathogenic when mutated (representing a high pathogenic hotspot). A score of 0.5 means that approximately 50% of the neighboring residues are pathogenic when mutated. Neighboring residues were considered pathogenic only if their pathogenicity was rated 4 or 5 according to the ACMG classification.

**
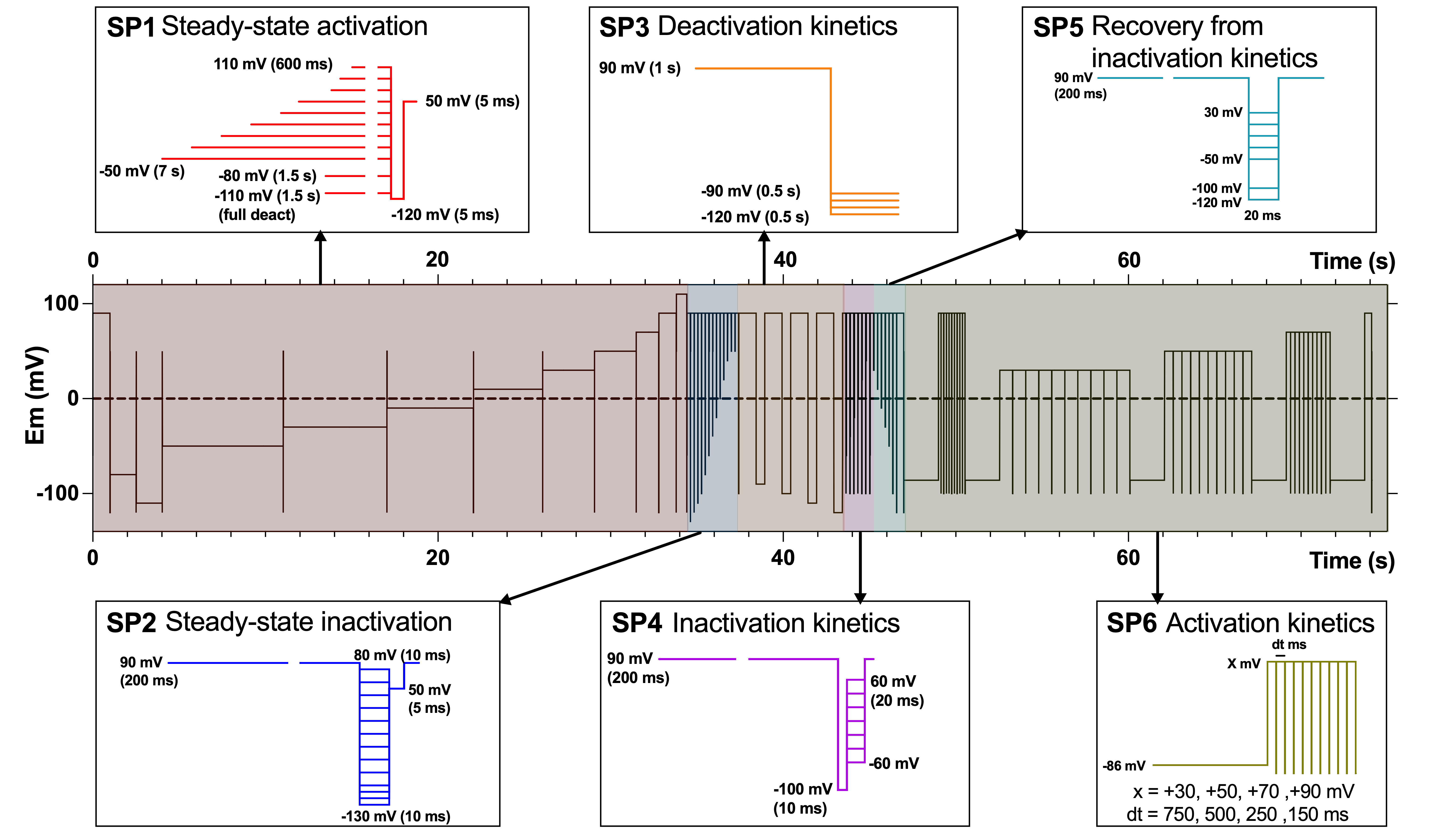
**

**Figure S8**. Six subprotocols from the fast-track protocol published for conventional patch-clamp ^3^ and adapted to automated patch-clamp system.


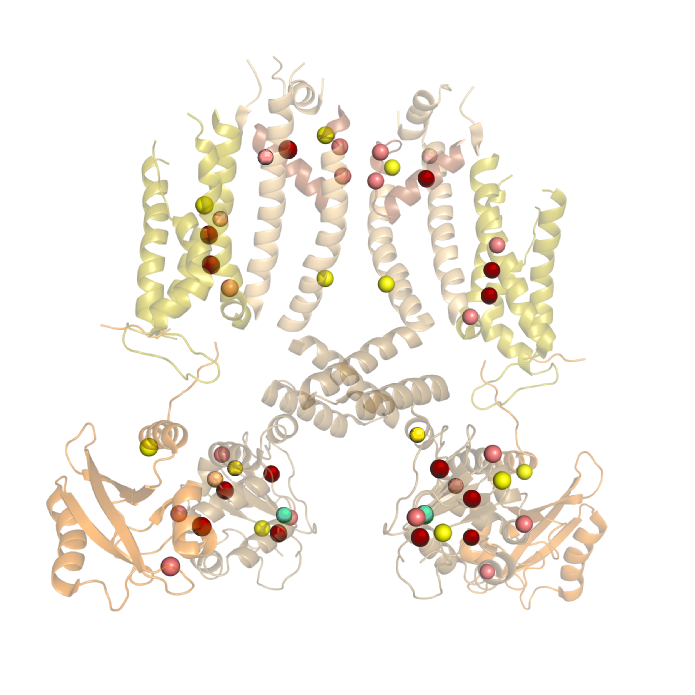


**Figure S9**. Distribution of the 24 ACMG variants investigated for which the full structural scoring is available. Green, yellow and red spheres represent the position of class 2, 3, and 4 & 5 ACMG variants, respectively. Two of the four subunits are represented here to facilitate visualization.

*References*

[1] Abo El Maaty MIA-E, Mervat M.; Abd Elwahaab, Marwa A. 3D graphical representation of protein sequences and their statistical characterization. In: Elsevier, ed. *Physica A: statistical mechanics and its applications*. ScienceDirect 2010: 4668-4676.

[2] Miller S, Janin J, Lesk AM, Chothia C. Interior and surface of monomeric proteins. *J Mol Biol* 1987; **196**: 641-656.

[3] Oliveira-Mendes B, Feliciangeli S, Menard M, Chatelain F, Alameh M, Montnach J, et al. A standardised hERG phenotyping pipeline to evaluate KCNH2 genetic variant pathogenicity. *Clin Transl Med* 2021; **11**: e609.

[4] Zamyatnin AA. Protein volume in solution. *Prog Biophys Mol Biol* 1972; **24**: 107-123.

[5] Higginbotham C. *Introductory biochemistry*: Creative commons.

[6] Simm S, Einloft J, Mirus O, Schleiff E. 50 years of amino acid hydrophobicity scales: revisiting the capacity for peptide classification. *Biol Res* 2016; **49**: 31.

[7] Kyte J, Doolittle RF. A simple method for displaying the hydropathic character of a protein. *J Mol Biol* 1982; **157**: 105-132.

[8] Hessa T, Kim H, Bihlmaier K, Lundin C, Boekel J, Andersson H, et al. Recognition of transmembrane helices by the endoplasmic reticulum translocon. *Nature* 2005; **433**: 377-381.

[9] Moon CP, Fleming KG. Side-chain hydrophobicity scale derived from transmembrane protein folding into lipid bilayers. *Proc Natl Acad Sci U S A* 2011; **108**: 10174-10177.

[10] Zhao G, London E. An amino acid "transmembrane tendency" scale that approaches the theoretical limit to accuracy for prediction of transmembrane helices: relationship to biological hydrophobicity. *Protein Sci* 2006; **15**: 1987-2001.

[11] Dunbrack RL, Jr. Rotamer libraries in the 21st century. *Curr Opin Struct Biol* 2002; **12**: 431-440.
